# Photoactivation in Chemotherapeutic Compounds for Cancer Treatment: Opportunities Beyond Photodynamic Therapy

**DOI:** 10.1155/bca/7161202

**Published:** 2026-03-08

**Authors:** Simon Ngigi Mbugua, Eunice Adhiambo Nyawade

**Affiliations:** ^1^ School of Chemistry and Materials Science, Technical University of Kenya, P.O. Box 52428-00200, Nairobi, Kenya, tukenya.ac.ke; ^2^ School of Mathematics and Physical Sciences, Jomo Kenyatta University of Agriculture and Technology, P.O. Box 62, Nairobi, 000-00200, Kenya, jkuat.ac.ke

**Keywords:** cancer, photoactivation, photocages, photochemotherapy, photodynamic therapy

## Abstract

Photoactivation is the stimulation or regulation of a chemical or a chemical process by utilizing light of specific wavelength that corresponds to an absorbance optimum of the agent being used and can penetrate into tissues. In cancer therapy, photoactivatable drugs utilize this phenomenon by allowing the temporal and spatial regulation of their cytotoxicity using irradiation. Therefore, in order to reduce the adverse effects of platinum medications, photoactivatable anticancer pharmaceuticals, which might be site‐activated in the tumour region, are a viable option. This paper summarizes different types of photoactivatable anticancer compounds that would produce an active version of a drug by the process of photouncaging. The mode of photoactivation and rationale for drug design are summarized. The effects of typical complexes on cellular pathways, photocytotoxicity and dark cytotoxicity are explored. When compared to traditional Pt(II) anticancer medications, photoactivatable anticancer compounds provide a number of benefits, including ability to overcome drug resistance, and in situ monitoring of drug accumulation and activation inside cells. This review also covers the design approaches, synthesis techniques, photoresponsiveness and antitumour effectiveness of various photochemotherapeutic compounds. Future developments and challenges in incorporating photoresponsive metal complexes are also covered. This detailed review aims at encouraging further thorough investigation into this intriguing area of study by offering a summary of current developments in the design and development of photoresponsive compounds for cancer treatment and future clinical prospects.

## 1. Introduction

The inability of anticancer metallodrugs to distinguish between normal and malignant cells is a significant disadvantage to a certain extent leading to systemic toxicity. On the other hand, photoresponsive metal complexes are a potential class of anticancer treatments with enhanced selectivity [1–3]. Light can regulate metal complexes’ toxicity, making them minimally intrusive and spatially selective, all under a remotely managed approach. One of the main objectives of drug delivery technology is to be able to manage medication dosage in terms of amount, location and time. Better control enhances therapeutic efficacy while reducing negative effects. Stimulated delivery systems have been presented as systems that respond to an external stimulus, such as temperature, pH, applied magnetics, electrical or light fields, ultrasound, light or enzymatic activity [4, 5].

Transition metal complexes have proven effective in antitumour phototherapies during the past few decades. In several domains, which include photoinduced ligand exchange or release, diverse excited state activity and adaptable biochemical features, they have demonstrated encouraging characteristics [6, 7]. Photoresponsive metallopolymer nanoparticles (MPNs), for example, exhibit improved hydrosolubility, prolonged blood circulation and elevated tumour‐specific retention when integrated into polymeric systems and nanostructures [8]. This results in a significant improvement in tumour treatment efficacy when in comparison with low‐molecule‐weight metallic complexes [8].

The current state of pharmaceutical treatments employed in cancer chemotherapy is significantly impacted by their harmful side effects and low selectivity. The majority of pharmacological molecules now on the market are not selective to the pathological sites, making it difficult to provide effective therapy at the targeted disease locations. Due to its special qualities, which include its minimally invasiveness and high spatiotemporal resolution, light‐mediated therapeutic strategies have lately become one of the most promising and effective tools for precisely controlling the activation of therapeutic reagents and imaging probes in vitro and in vivo [9].

Thus, in addition to discovering new, effective, targeted antitumour drugs, it is crucial to come up with creative ways to provide cancer chemotherapy with a more focused, targeted impact. An alternative that has long been of interest to researchers is the application of different internal triggers (such as pH, redox environment and enzymatic processes) or external triggers (such as ultrasound, magnetic field and light irradiation) for the on‐site induction of stimuli‐sensitive drug compounds or drug delivery systems [10]. Out of all the possible physical stimuli, light stands out for a number of reasons, including its exact control over the wavelength and amount of radiation, as well as its interactions with biological systems [11].

**SCHEME 1:**
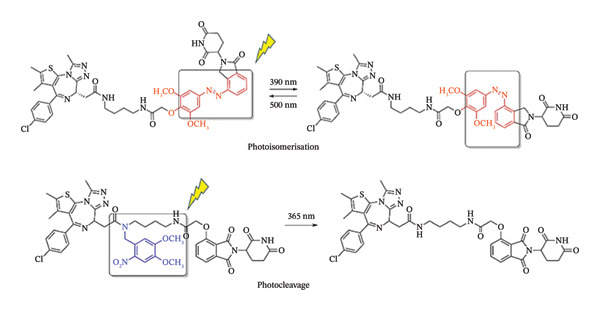
Photocaged vs. photoswitchable pharmacological agents showing the respective photoactivated transformations. Adapted from [13]. Cancers, published by MDPI, 2021.

In this regard, the area may also profit from the instruments already existing for photodynamic treatment (PDT). Light activation can target a range of malignancies since it is not influenced by the characteristics of the environment within the tumour. Following the approval of the first PDT regimens in the 1990s, light‐activation‐based techniques were put into clinical use [12]. Two light‐activation‐based strategies also surfaced. As outlined by Scheme 1, photocaged vs. photoswitchable pharmacological agents are described with the respective photoactivation mechanisms. These mechanisms utilize photoswitches and photoremovable protective groups (PPGs), or “photocages” [13], an area is also referred to as photopharmacology.

Molecules that are photocaged are inactive in the absence of light and become physiologically active only upon exposure to light [14]. The caged molecule can be released with spatiotemporal control thanks to this light activation [15]. Another way that photocaging differs from PDT is that it is not dependent on oxygen, whereas photosensitizers (PSs) often need a high oxygen concentration to function [16, 17]. The cage and the caged molecule are the two halves of a photocaged complex. The drug is released from the caged molecule when it absorbs a photon of light at the right wavelength, making the caged molecule physiologically active. This approach is referred to as photochemotherapy (PCT) when used in cancer therapies [18, 19].

Both photocaging and photoswitchable strategies are feasible for small compounds, where photoactivatable parent drugs are made by structural alterations, or for more sophisticated drug delivery systems, photoactivatable units are strategically attached into the framework [20, 21].

## 2. Comparative Overview of PSs in Phototherapy

There are primarily three forms of phototherapy: photodynamic therapy (PDT), photothermal therapy (PTT) and photoactivated chemotherapy (PACT). The three main elements of PDT and PACT are a PS, exposure to a certain wavelength of light and the presence of molecular oxygen (or another oxidizable substrate). In each instance, the PS absorbs a photon, undergoes photochemical processes that produce reactive oxygen species (ROS) and is transformed into an excited state (first singlet, then triplet via intersystem crossover). Target cells (tumour cells or bacteria) suffer oxidative damage as a result, which causes cell death or inactivation.

PDT is now used to treat select malignancies that are easily accessible with lamps or optic fibres, such as some forms of head and neck, cutaneous, bladder, lung, oesophageal cancers and others. It offers significant benefits, including temporal and spatial manipulation. It is additionally being intensively investigated for use in other contexts, including for the cure of bacterial and fungal illnesses that are immune to other drugs [22].

**FIGURE 1:**
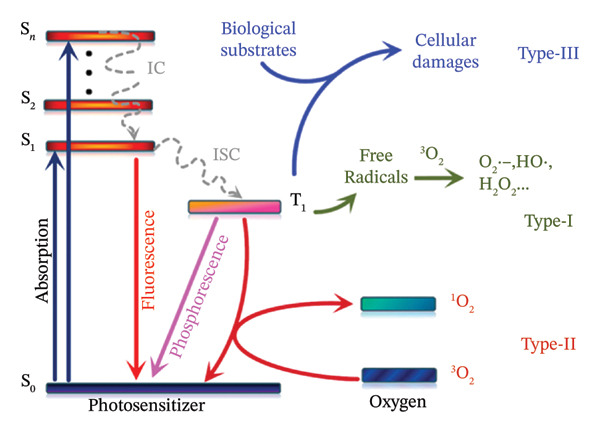
Jablonski’s diagram for the principle of Type I, Type II, and Type III PDT. S_0_, the singlet ground state; S_1_, the first excited singlet state; S_2_, the second excited singlet state; S_n_, the *n*th excited singlet state; IC, internal conversion; ISC, intersystem crossing. Reprinted with permission from [23] Copyright (2022), Elsevier.

Two primary PDT mechanisms of action (Type I, which includes the generation of ROS, and Type II, in which ^1^O_2_ directly triggers the damage) depend on the PS’s ability to change oxygen from its triplet ground state to its singlet excited state. PDT typically entails the injection of a PS, which produces cytotoxic species when exposed to oxygen and is triggered by visible light. These mechanisms are shown in Figure 1 [23]. Utilizing e/H^+^ transfer mostly from PS ^∗^(T_1_) states, a Type‐I PS produces oxygen‐free radicals such as the hydroxyl radical (OH^−^) and superoxide radical (O_2_^−^).

Conversely, a Type‐II PS produces singlet oxygen (^1^O_2_) and PS (S_0_) by triplet‐triplet annihilation, which is fuelled by electron spin exchange between PS ^∗^(T_1_) and triplet oxygen (^3^O_2_). In other words, oxygen is necessary for Type‐I and Type‐II processes to produce ROS as intermediates. Oxygen is required for photosensitization to occur but solid tumours thrive in a hypoxic environment. This has a big impact on how well photosensitive medications kill tumour cells. As a result, much effort has been put into preventing PS reliance on oxygen‐dependent PDT [24].

Tumours, specifically in the inner core region, are frequently hypoxic. As mentioned above, it would be quite intriguing to create a light‐mediated approach that is independent of oxygen. In this way, light can have other effects besides singlet oxygen production. It is known that chemical bonds may either cleave or reorganize when exposed to light. In fact, a compound’s functionality may be changed by enclosing some of its functional groups in a photoremovable group. Then, exposure to light can regulate the original compound’s release.

A number of medications and drug candidates have been effectively released when exposed to light using photocages. These include inhibitors of carbonic anhydrase II, aspirin, ibuprofen, ketoprofen and a few anticancer drugs including floxuridine, paclitaxel, tegafur, doxorubicin and a photoreactive DNA intercalator [25]. A cleavable singlet oxygen bridge was previously used to attach the anticancer drug candidate combretastatin A‐4 to a porphyrin PS in a recent combination of photocaging and singlet oxygen production [26].

On the other hand, photothermal treatment uses metal complexes to convert light into thermal energy through nonradiative relaxation pathways. This process is referred to as the photothermal effect. Transition metal complexes represent a viable alternative for anticancer phototherapy, since they exhibit high toxicity towards cancer cells through photoreduction and photosubstitution processes. Generally, a sequence of photochemical reactions or photothermal effects are started to kill tumour cells when low toxicity metal complexes precisely concentrate in tumour areas through blood circulation and are triggered by light of specified wavelengths. For in vivo applications, photoresponsive metal complexes continue to present challenges. This is because of their poor stability in physiological settings and adverse metabolic/biodistribution. Additionally, metal complexes are quickly removed from the circulation and cannot effectively accumulate at tumour sites.

Furthermore, metal complexes may have hazardous adverse effects and are often not biocompatible. Thus, the development of metal complex‐containing materials that tackle these issues for the treatment of tumours in vivo is extremely important. In this regard, metallopolymers that are produced by integrating or incorporating metal complexes into polymeric scaffolds show promise for use in living organisms [27, 28].

## 3. Photoactivation UV Responsive Photocages

Light of various wavelengths is a perfect stimulus with noninvasive qualities and spatiotemporal accuracy. Generally, a photocleavable functional group that inhibits the action of therapeutic molecules is activated by a light beam, particularly in the short wavelength UV range. This allows for carefully regulated drug release in the target microenvironment.

**SCHEME 2:**
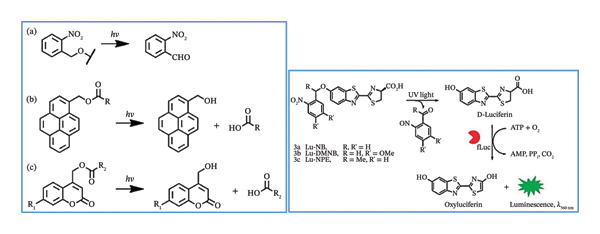
UV light‐mediated uncaging strategies: (a) o‐nitrobenzyl, (b) pyrenylmethyl ester, (c) coumarinyl ester, and UV light‐induced release of D‐luciferin from the ONB photocaged D‐luciferin derivatives. Reprinted with permission from [30]. Copyright 2015. American Chemical Society.

Numerous other photocaged compounds have been developed for spatiotemporal regulation of physiological events, real‐time cell transport surveillance and regulated discharge of active compounds in vitro and in vivo. Examples of these molecules are o‐nitrobenzyl (ONB), pyrenylmethyl ester and coumarinyl ester [9, 29] which are shown in Scheme 2 that outlines the UV light‐mediated uncaging strategies for these compounds.

Photocaged nitrobenzene ligands, for example, have been utilized to study the firefly luciferase (fLuc) bioluminescence reporter in real time, both in vitro and in vivo. To do this, a series of stable and effective photoactivable D‐luciferin probe molecules were developed by several researchers. As illustrated in Scheme 2, the inactivation of D‐luciferin’s 6‐hydroxy unit by various nitrobenzene ligands was responsible for initially preventing D‐luciferin’s bioactivity [30, 31].

These photocaged devices, which exhibit powerful bioluminescence imaging in living mice, were quickly released to restore the activity of D‐luciferin under UV light irradiation at 365 nm [30].

Using low‐energy radiation, McCoy et al. created a molecular approach for light‐triggered drug administration of several kinds of drugs [32]. The amount of applied light accurately controls the drug dosage. The molecular unit functions as a dosage device at the molecular level when combined with the proper polymer scaffold [32]. Using a light‐controlled drug liberation reaction, the high degree of control that can be applied to light imposed to a molecule in terms of wavelength, duration, intensity and location can be utilized to give control over the amount of drug released (the dose), the exact moment of the release event and where it occurs [32].

Significantly, this control may function at the single molecule level, enabling conjugate‐incorporated media to function as molecularly controlled drug dosage devices [33]. Derivatives of 3,5‐dimethoxybenzoin (3,5‐DMB) have been employed in the past as protective groups in chemical synthesis, which may be detached by applying light [34]. This method of protecting molecules having carboxylic acid or secondary amine functional groups involves reacting them with 3,5‐DMB to produce esters or carbamates, respectively.

**SCHEME 3:**
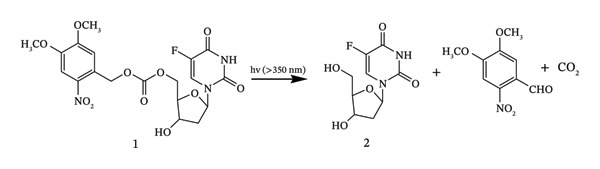
Photolysis of **1** with long‐wavelength UV light rapidly released **2** in solution. Adapted with permission from [35]. Copyright (2020), Elsevier.

Wei et al. synthesized a photolabile derivative (1) of the anticancer medication 5‐fluorodeoxyuridine (**2**) as a model prodrug [35]. Using long‐wavelength UV light to photolyse Compound **1**, Compound **2** was quickly liberated into solution (Scheme 3). Compound **1** by itself is not toxic to cells, but when combined with UV light (*λ* = 350 nm), it significantly inhibited the proliferation of cells. Long‐wavelength UV light photoactivated the nontoxic, photolabile Prodrug **1** into the toxic Drug **2**, resulting in a strong suppression of cell development.

## 4. Infra‐Red Responsive Photocages

The traditional approaches of light‐mediated theranostics, while initially successful, are mostly focused on short‐wavelength light (UV or visible light), which typically has a number of undesirable side effects. These involve the possibility for phototoxicity to healthy tissues, the restricted depth of tissue penetration and inevitable light absorption and dispersion [36].

Due to their exceptional optical qualities, the 4,4‐difluoro‐4‐bora‐3a,4a‐diaza‐s‐indacene (BODIPY) and its analogues are widespread fluorescent dyes that have become utilized extensively in bioimaging, disease diagnostics and PDT [37, 38]. They exhibit long nanosecond‐scale fluorescence lifespan, strong molar absorption coefficients, intense fluorescent emissions with comparatively narrow emission bandwidths and photostability, among other qualities. With these characteristics, Goswami et al. have reported that meso‐BODIPYs might serve as photocages as well [39]. They developed a computationally searching technique to show that, under green light irradiation (515–553 nm, 500 W), the meso‐position (C8) on BODIPY (II‐1, II‐2, Figure 2(a)) was prone to create carbocation with a low‐lying excited state to liberate acetic acids.

FIGURE 2BODIPY‐based photocaging structure. (a) Basic structure of meso‐methyl BODIPY‐based photocages. (b) BODIPY‐based photocages with diverse subcellular sites. (c) BODIPY‐based photocages with enhanced water solubility. Reprinted with permission from [40] Copyright (2023), Elsevier.

**Figure:**
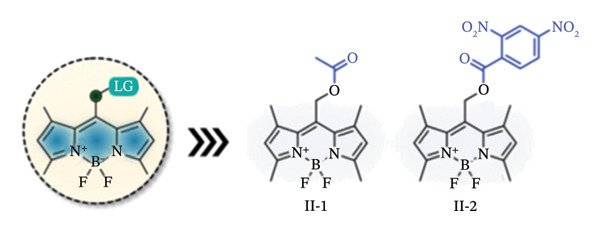
(a)

**Figure:**
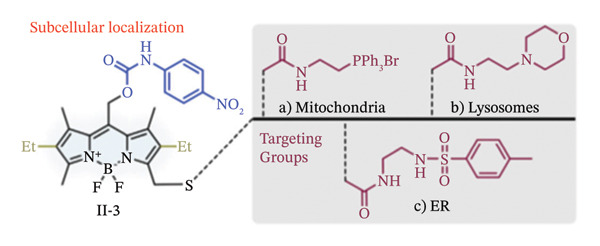
(b)

**Figure:**
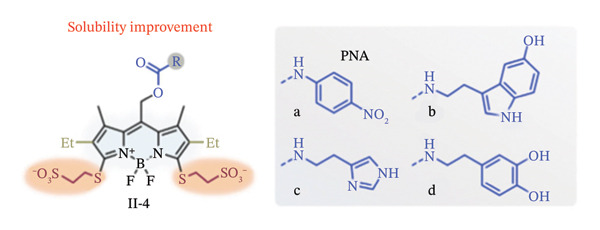
(c)

Using this platform, they coupled several organelle‐targeting groups to the a‐methyl position in order to accomplish subcellular photorelease (II‐3, Figure 2(b)). Live‐cell fluorescence was seen in comparable organelles with the release of p‐nitroaniline (PNA) under the excitation light (545 nm, 42mWcm}2). These findings led to the development of a novel medication delivery method based on the spatiotemporal regulation of photouncaging [41].

Further optimization of the structures of BODIPY has been attempted by the addition of hydrophilic sulfonic acid groups without affecting their spectroscopic or photoreaction characteristics in order to increase the water solubility of such BODIPY‐based photocages [41]. In this regard, sulfonates with long linkers have been connected at different sites while electron‐donating groups (EDGs) were added to the 2,6‐positions of the BODIPY structure (II‐4, Figure 2(c)). Increased solubility and light‐dependent release of dopamine uncaging in neurons were the outcomes of this chemical designs. Thus, the cellular localization of water‐soluble BODIPY‐based photocages was completely regulated, while keeping the photoreaction properties unaffected.

## 5. Metal Complexes in Photocages

It has also been suggested that the use of light to treat cancer, which has significantly contributed to the clinical development of PDT, represents an intriguing advancement in the study of metal‐based anticancer drugs that do not require oxygen to function, like PSs. There are numerous publications on the utilization of photocaged organic molecules, but only few instances of the specific release of intact (organo)metallic compounds triggered by light. It is true that some complexes have light‐induced reactions in which the metal centre participates. This characteristic was utilized to create metal‐based photocages in addition to increasing their cytotoxicity. Cytotoxicity may also be induced by the release of metal ions or a labile metal complex, as demonstrated by employing a photolabile o‐nitrophenyl moiety [42].

In the initial stages of their production, photocages were mostly made of organic substances. In 1978, an ONB group was used in a pioneering instance of photocaging, which released ATP in response to UV light [43, 44]. Metal coordination complexes have been used as photocages more recently. To achieve uncaging, metal complexes go through a convoluted series of electronic transitions [45, 46]. The molecule may excite an electron from the HOMO (^1^GS) to the LUMO upon photon excitation [47]. The metal‐to‐ligand charge transfer (^1^MLCT) state is the name given to this singlet excited state.

Subsequently, the excited electron can reach a triplet excited state (^3^MLCT) by undergoing an intersystem crossover and spin inversion. The electron’s destiny inside the ^3^MLCT is dependent upon the energy levels of the accessible decay routes. The metal complexes may undergo ligand cleavage and shift into the triplet metal cantered state (^3^MC) if the ^3^MC state is thermally accessible. The ^3^MC state will then relax back to the ground state via phosphorescence or a nonradiative decay process if its energy is too high in comparison to the ^3^MLCT state [48].

## 6. Ruthenium (II) Metal‐Based Photocages

Many low‐spin d^6^ transition metal compounds, such as Mn(I), Re(I), Fe(II), Ru(II), Os(II), Rh(III), Ir(III) and Pt(IV), are photoactive [49]. Certain complexes may exhibit phosphorescence decay from a triplet excited state and have long‐lived excited states, based on the kinds of ligands. Others may suffer redox reactions resulting from photoinduced metal and/or ligand electron transfer, or they may undergo metal–ligand bond weakening in singlet or triplet excited states, hence promoting ligand release [50].

Historically, photocaging has been used on functional groups present in inactive compounds such as carboxylic acids, alcohols and amines [51, 52]. UV radiation is necessary for the majority of photoremovable organic protective groups to uncage, which negatively affects biological systems by causing irreversible photodamage. Moreover, organic groups do not shield functional groups which are unprotectable by organic molecules. These groups include nitriles and aromatic heterocycles, which are crucial “warheads” that engage in direct interactions with heme and thiolates in the active regions of protein targets [52].

For caging applications, transition metal fragments are appealing, particularly complexes that attach to functional groups that are unprotectable by organic molecules. Therefore, an alternative method for photocaging bioactive compounds is provided by metal coordination. Metal‐based photocaging makes it easy to release bioactive molecules when exposed to low‐energy light since transition metal complexes frequently show strong charge transfer absorption in visible wavelengths [48, 53], and the bond between the metal and its related ligand is nearly always less stronger compared to an organic sigma bond [54]. One may adjust the photochemistry to achieve drug release throughout a wide spectral range, from the visible to near‐infrared (NIR) frequencies, by adjusting accessory ligands.

In this regard, metal‐caged compounds are intriguing chemical substances for biological investigation and applications because they can be spatiotemporally controlled to free them selectively. Metal‐based photocaging is currently being used to create innovative light‐activated therapies in addition to other fundamental research uses [13, 55].

Photocages made of ruthenium(II) polypyridyl complexes have been synthesized and reported by several researchers. Ru(II) complexes’ octahedral shape allows for excited state ligand dissociation, a property that is not available for square planar Pt(II) such as cisplatin [56]. Ru(II) polypyridyl complexes can absorb in the visible spectrum and are generally thermally inert in aqueous solutions [57]. Some Ru(II) complexes concentrate at tumour cells because of their ability to imitate iron binding [58, 59] which is a desirable feature for drug administration. After light is absorbed, the thermal population of 3d‐d states transitions from a triplet ^3^MLCT state leads to ligand dissociation in many Ru(II)‐based polypyridyl complexes.

Metal complexes made of ruthenium are highly valued for their facile singlet oxygen generation and significant absorption in the visible spectrum [60]. They may be adjusted for certain therapeutic uses and frequently show high photostability [61]. Because of their great degree of tunability, ruthenium(II) polypyridyl complexes may be designed to absorb light in the visible (400–600 nm) or even NIR spectrum by modifying their ligands. This is the case for [Ru(bpy)_2_L]^2+^ complexes (where bpy = 2,2′‐bipyridine), whereby, when exposed to radiation, they can release bioactive ligands. The ability to absorb light is beneficial for deep tissue penetration, which is a significant drawback for many PDT medicines. As effective light‐responsive agents, Ru(II) complexes proceed through MLCT or photoinduced ligand dissociation processes.

A class of inhibitors that consist of nitrile‐containing compounds reacts with the thiolates found in the active site of cysteine proteases thereby inactivating them [62–64]. The nitrile functions acts as the “warhead” of these compounds, forming a covalent link with the target protein [65, 66]. For instance, the nitrile‐based cathepsin K inhibitor odanacatib, which has progressed to Phase III clinical trial for the treatment of osteoporosis, binds to the target enzyme’s active site and decreases bone resorption [67, 68]. It has been established that cysteine proteases play crucial roles in the emergence of cancer [69, 70]. Cysteine protease suppression, therefore, has anticancer effects via preventing metastatic growth and invasion, as well as by preventing enzyme‐substrate interactions [71]. Cysteine protease inactivation only happens under light irradiation since nitriles may be securely coupled to Ru(II).

In this regard, Ru(II)‐based protecting groups have been employed to cage known nitrile‐based cysteine protease inhibitors in order to modulate cysteine protease inhibitions both spatially and temporally with the caged antagonists exhibiting the capacity to be liberated upon exposure to light.

**FIGURE 3:**
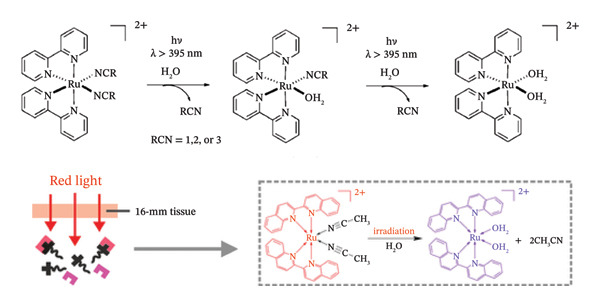
Schematic representation of photoinduced ligand exchange in Ru(bpy)_2_‐caged nitrile‐based cysteine protease inhibitors. Reprinted with permission from [52] Chemical communications (2028), Royal Society of Chemistry.

Each of the antagonists has an electrophilic nitrile group that can interact with cysteine proteases’ target sites’ thiolate to block the enzyme. The photochemical characteristics of their Ru(II)‐caged complexes are typically similar. Figure 3 indicates the process of photoinduced ligand exchange in Ru(bpy)_2_‐caged nitrile‐based cysteine protease inhibitors. In this mechanism, they remain stable in aqueous media under dark conditions. However, when exposed to visible light, they successfully discharge the caged drugs, usually releasing both of the two equivalents (Figure 3) [52]. All caged compounds display a substantial increase of cysteine protease inhibition when exposed to light, with each light‐activated complex exhibiting a stronger effect on cysteine protease inhibition compared to inhibitor on its own [52].

Treatment of [Ru(bpy)_2_ [1]_2_]^2+^ with light irradiation results in the B32‐fold improvement of the inhibition of cysteine protease papain in comparison to dark experiments [52]. Papain‐based control studies using [Ru(bpy)_2_(MeCN)_2_]^2+^ rule out the potential for enzyme inhibition brought on by other photolysis products, suggesting that the Inhibitor 1 produced in response to light irradiation is the source of enzyme inhibition [52].

**FIGURE 4:**
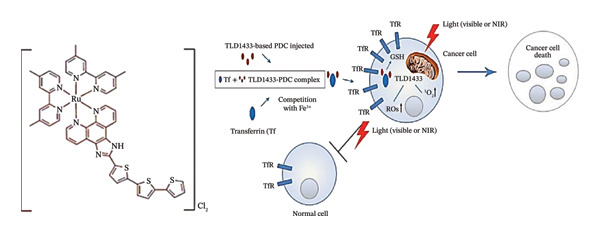
Chemical structure of TLD‐1433 and its NIR‐activated mechanism of action. Reprinted with permission from [72] Copyright (2022), Elsevier.

The luminescent 4d^6^ Ru(II) complex TLD1433 (Ruvidar®, also referred to as Rutherrin) (Figure 4), which has undergone clinical trials for the management of bladder cancer, is activated by green light causing it to produce ^1^O_2_. It is water soluble and characterized by high ROS generation. It is known to preferentially accumulate in bladder tumours targeting transferrin receptors (TfR).

In contrast to TLD1433’s PDT effects, 5d^6^ Pt(IV) complexes frequently break down directly to produce cytotoxic species even in the absence of oxygen [73]. This property makes them potentially useful in PACT, which includes phototherapy for hypoxic malignancies.

Modifications to the structure of tris N^N Ru complexes are widely recognized approaches where, for example, two complexes with the structure [Ru(phen)_2_(7R,8R‐dppz‐)]^2+^ (R = OH or ‐OMe) were reported by the Gasser group [74]. Intense phosphorescence and high ^1^O_2_ quantum yields were demonstrated by both complexes, but their cellular accumulation and toxicity were different. It is possible to fine‐tune PDT characteristics through modifications of the intercalating groups, as demonstrated by the various toxic effects reported for each complex before and after irradiation (at 420 or 800 nm for monolayer complexes or spheroids, respectively) [75]. Different classes of these complexes frequently exhibit uneven trends, which can lead to misunderstandings. For instance, it was once believed that the presence of bulky groups near the metal centre increased photodissociation and consequent toxicity. Nevertheless, studies conducted under acidic and basic conditions revealed that deprotonation decreased dissociation and toxicity in bis‐heteroleptic N^N complexes with ionizable groups positioned to put a strain on the coordination sphere [76]. Comparing deprotonated complexes to their protonated counterparts, higher ROS quantum yields were observed, indicating that phototoxicity in the complex is caused by ROS rather than photodissociation [76].

## 7. Ruthenium PACT Compounds

Microtubules are crucial for the separation of double chromosomes prior to cell division during mitosis. Because of this characteristic, they are a good target for new chemotherapeutic drugs, which selectively destroy rapidly dividing cells. Paclitaxel is one of the most effective microtubule targeting drugs used in clinical settings. It is utilized for the treatment of several types of nonsmall cell lung cancer, ovarian cancer and breast cancer. Paclitaxel causes apoptosis and cell death by interfering with the dynamics of microtubules. However, adverse effects from paclitaxel‐based treatment are common and involve neurotoxicity, neutropenia and irregular heart rhythms, among other things. Furthermore, the clinical effectiveness of paclitaxel chemotherapy is limited due to cancer cell tolerance. Therefore, an important field of cancer research involves developing alternative microtubule‐targeting drugs or techniques.

When compared to their ability to function as PSs, the field of Ru(II)‐based photocages is far less established. There have been important discoveries in the recent past that show the wide range of beneficial applications for this technology [77–80]. The first instance of uncaging a neurotransmitter from a Ru(II) complex was accomplished in 2013 by the Etchenique group (Figure 5(a)) [81, 82]. A Ru(II) photocage complex demonstrating the first instance of light‐activated protease inhibition was reported in 2011 (Figure 5(b)) [81].

FIGURE 5Ru(II)‐based photocage reactions. Adapted from [81].

**Figure:**
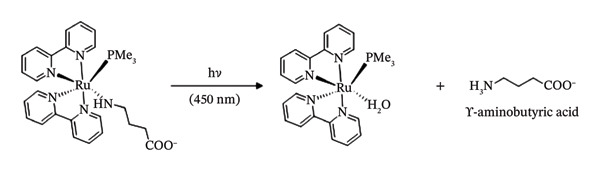
(a)

**Figure:**
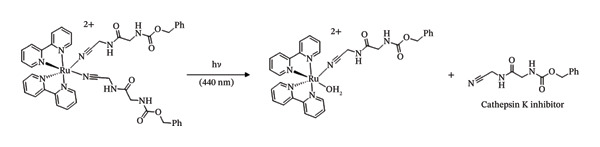
(b)

**Figure:**
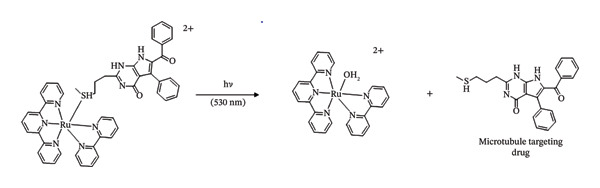
(c)

The intense absorption of visible light into singlet ^1^MLCT states, which undergoes intersystem crossing with almost 100% yield to fill corresponding triplet MLCT (^3^MLCT) states, is a key characteristic of Ru(II) complexes [83, 84]. When a ligand is coordinated with an extended π‐system that has a ligand‐cantered π‐π ∗ state that is below the ^3^MLCT state, long excited‐state lifetimes (20–50 μs) are generated [85, 86]. The production of singlet oxygen (^1^O_2_) is the mechanism of action for the majority of Type II PSs used in clinical PDT.

Significant progress has been achieved subsequent to these foundational works. A Ru(II) photocaged complex irradiated with green light has been shown to release a drug that targets microtubules, in contrast to the previously mentioned examples which were uncaged by the use of blue light of higher energy [87, 88].

Moreover, this derivative of a photocaged system was the first Ru(II) PCT agent to be triggered in an in vivo mouse model—a unique outcome that was crucial to the advancement of this discipline (Figure 5(c)) [88].

Moreover, complexes with sterically hindering ligands that deviate from the ideal octahedral structure in the arrangement around the Ru(II) centre produce lower energy metal‐centred states of antibonding character that can be populated from ^3^MLCT states [89, 90]. This makes ligand disengagement easier and is a notion that is utilized in PCT. Ru(II)‐based complexes with dual functionality that are accessible with low‐energy illumination have been reported by several researchers. These complexes can be used as drug models since they can produce ^1^O_2_ through photosensitized photosynthesis and cause aromatic heterocycles to photodissociate [81]. A crucial component of this dual action PDT/PCT drug was the π‐expansive ligand 3,6‐dimethylbenzo[i]dipyrido[3,2‐a:2′,3′‐c] phenazine (Me_2_dppn), which is composed of a phenanthroline core linked with a diaminonaphthalene moiety.

Significantly, it was demonstrated that effective, light‐activated cell killing in in vitro models of triple‐negative breast cancer was achieved through the combination of PCT and PDT with a Me_2_dppn‐containing Ru(II) complex [81]. In these 2D and 3D culture models, related complexes that underwent PCT or PDT alone exhibited little to no cytotoxicity, indicating that combined action PCT/PDT generates and enhances the effectiveness of Ru(II) compounds against cancer cells.

Compared to conventional chemotherapy, PDT has been demonstrated to cause less systemic damage in cancer patients because light is administered precisely to the tumour environment. Nonetheless, PDT and PACT vary fundamentally from one another. Through the energy or electron transfer of the excited prodrug molecule to the O_2_ present in the irradiated tumour tissues, a light‐irradiated PS in PDT produces cytotoxicity [91, 92]. PDT is therefore very effective in cancers with adequate oxygenation, but it frequently fails in hypoxic tumours because low O_2_ concentrations prevent oxidative stress from being produced by photolysis [93–96].

Quite the reverse, PACT relies on an O_2_‐independent bond cleavage photoreaction for its light stimulation process, which means that PACT should theoretically enable the development of anticancer phototherapies that do not rely on intratumoural oxygen levels and can thus be used for treating hypoxic tumours. An important goal in oncology is to come up with novel therapies that are safe in the dark, that upon irradiation with light concentrate on a well‐established cancer target and that remain effective under hypoxic environments. This is because oxygen‐deficient tumours are highly challenging to treat by PDT, radiation therapy, chemotherapy or immunotherapy [97, 98]. Studies have examined and reported the use of [2](PF_6_)_2_ as a novel PACT complex that releases rigidin Compound 1 that targets microtubules when exposed to green light [99].

**SCHEME 4:**
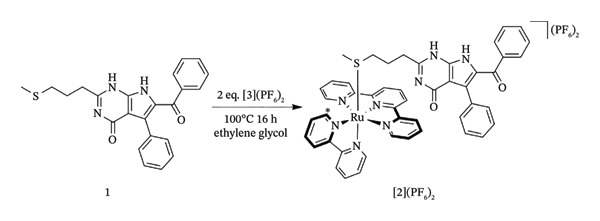
Synthesis of [**2**] (PF_6_)_2_. ^∗^The A6 proton of the 2,2′‐bipyridine (bpy) ligand [88].

The studies provided quantitative phototoxicity experiments in vitro, showing effective light activation at low O_2_ concentrations under normoxia (21% O_2_) and hypoxia (1% O_2_) [88]. Furthermore, it is demonstrated for the first time in 3D tumour spheroids and human lung cancer (A549) xenografts in nude mice the light activation of a ruthenium‐based PACT complex. Thioether‐containing microtubule Inhibitor **1** was reacted with two equivalents of [Ru(tpy) (bpy) (OH_2_)](PF_6_)_2_ ([3](PF_6_)_2_ (Scheme 4) to create the complex [Ru(tpy) (bpy) [1]](PF_6_)_2_ ([2](PF_6_)_2_) [88].

When [2](PF_6_)_2_ was dissolved in acetonitrile and exposed to green (*λ*_irr_ = 530 nm) light in an inert dinitrogen environment, the wavelength of absorption at 452 nm increased significantly, and the maximum of the singlet metal‐to‐ligand charged transfer (1MLCT) band was slightly shifted from 452 to 454 nm [88]. When the solution was subjected to light irradiation, mass spectrometry (MS) revealed peaks at *m*/*z* = 266.4 corresponding to [Ru(tpy)‐(bpy) (CH_3_CN)]^2+^ (calcd *m*/*z* = 266.1). In contrast, a strong signal was seen at *m*/*z* = 447.0 corresponding to [2]^2+^ (calcd *m*/*z* = 447.1). This indicated that Ligand **1** was photosubstituted by a solvent molecule when exposed to green light [88].

## 8. A Comparative Analysis of the Biocompatibility, Cytotoxicity and Photoresponsiveness of Ruthenium (II) and Platinum‐Based Complexes

The photoresponsiveness, cytotoxicity and biocompatibility of these photocaged ruthenium (II) and platinum complexes can be summarized in Table 1. This outlines the factors in terms of modes of action, wavelengths of interest, biocompatibility and delivery profiles among others.

**TABLE 1:** Summary comparison of ruthenium(II) and platinum‐based complexes.

Factor	Ru(II) complexes	Pt complexes	References
Photoresponsiveness	Strong (visible/NIR light possible); MLCT transitions ideal	Moderate (mostly UV, some visible‐responsive Pt(IV) systems)	[100, 101]
Photoactivation mechanism	Ligand dissociation, ROS generation or prodrug release	Photoreduction (Pt(IV ⟶ II)), ligand photodissociation	[102, 103]
Activation wavelength	Tunable (300–770 nm; some NIR designs)	Typically UV (320–400 nm); NIR‐active Pt complexes still emerging	[104, 105]
Excited‐state lifetime	Long‐lived (microseconds); enhances photocleavage efficiency	Shorter lifetimes; less efficient photochemistry unless engineered	[106]
Cytotoxicity (dark)	Generally low; stable in biological conditions	Pt(IV) prodrugs have low dark toxicity; Pt(II) photocaged forms may be more toxic	[107, 108]
Cytotoxicity (light)	High cytotoxicity postactivation; DNA/RNA damage, ROS	High; photoactivated Pt(II) forms form DNA crosslinks or adducts	[109, 110]
Selectivity and control	High spatiotemporal precision; often used with targeting ligands	Reasonable control with prodrug design; UV limits precision in tissues	[104]
Biocompatibility	High; good aqueous solubility and tunable pharmacokinetics	Moderate to good; Pt(IV) prodrugs designed for improved compatibility	[111, 112]
Theranostic capability	Strong luminescent for imaging and therapy	Limited nonluminescent unless modified	[113, 114]
Resistance profile	Less prone to cisplatin‐type resistance mechanisms	Pt(IV) can evade some resistance; Pt(II) complexes still face efflux, detox issues	[115, 116]
Delivery enhancements	Often integrated into liposomes, nanoparticles, conjugates	Similar strategies used; lipophilic cages can aid delivery	[111, 117]

Photorelease therapeutics such as NAMPT inhibitors, microtubule inhibitors, metabolic inhibitors and enzyme inhibitors have excellent preclinical promise in a variety of Ru(II) polypyridyl complexes (cells and some animal models). These are only preclinical; although the area is active, human trials have not yet been implemented. Clinical challenges with translation have already been discussed in other reviews [118]. These include pharmacology issues, safety concerns, formulation challenges, tissue penetration of activating light and administration.

## 9. Structure–Activity Relationships of Ruthenium(II) and Platinum Photocages

As would be expected, the cytotoxic profile, tumour‐targeting potential and photoactivation effectiveness of ruthenium(II) and platinum‐based photocaged complexes are all significantly influenced by the ligand structure. Modifications to the ligand type, coordination geometry or metal centre can impact the complexes’ susceptibility to photoactivation, therapeutic qualities, including subcellular targeting, light absorption, activation mechanism, biological interactions, solubility and stability. These factors can be utilised to carry out rational drug design for these complexes by understanding the structural, electronic and steric factors that affect the activity of these complexes.

A longer wavelength (visible/NIR light) can be used for activation with electron‐donating ligands since electron‐donating ligands reduce the MLCT energy [119]. Triplet‐state durations and ROS production are increased by π‐accepting ligands, such as phenanthroline, which improve intersystem crossing [120]. Through destabilization of the ground state, bulky ligands can hasten ligand dissociation. Factors like trans‐influence acts to weaken bonds which are directly opposite to a trans directing ligand, which can hasten ligand dissociation. Photoactivation can be accelerated by substituting monodentate ligands with bentate ligands, which frequently result in more stable complexes that are slower to photodissociate [79]. For instance, substituting 4,4′‐dimethyl‐2,2′‐bipyridine for one bpy in [Ru(bpy)_3]^2+^ changes the absorption maxima and improves photolability [121].

In platinum (IV) complexes, for example, the activation of the axial ligands requires photolabile groups such as nitrophenyls or azides. By enhancing orbital overlap, flexible ligands may enable more effective photoreduction. Steric effects can be enhanced by adding aromatic rings or halogens, which can improve spin–orbit coupling and increase photoreactivity [122]. The structure activity relationships are briefly summarized in Table 2, which addresses the effects of various functionalities like steric effects, ligand geometry and electronic effects among others.

**TABLE 2:** Structure‐activity relationships of ruthenium(II) and platinum photocages.

Structural change	Effect on photoactivation	Effect on cytotoxicity	Effect on targeting
Presence of electron‐donating groups	Reduction in activation energy	Increase or decrease (depends on target)	No direct effect
Steric effects around metal centre	Increases ligand photodissociation by destabilizing the ground state	Enhances cytotoxicity (if release is cytotoxic)	May affect delivery
Presence of targeting moieties	No effect	No effect	Enhancement of targeting specificity
Use photolabile axial ligands in Pt(IV) complexes	Greatly increases rates of photoreduction	Raises cytotoxicity under light	May improve binding to DNA nucleobases
Presence of intercalating ligands	Increases DNA interactions	Enhances dark toxicity	Improves nuclear targeting

Table 3 summarizes the structural and ligand factors that influence photoactivation efficiency, cytotoxicity and tumour‐targeting ability, with the corresponding electronic effects.

**TABLE 3:** Electronic effects structure‐activity relationships.

Structural modification	Electronic effect
Electron‐donating groups on ligand groups	Lowers MLCT transition energy resulting to a red‐shift absorption making the complex more responsive to visible light [123].
π‐extended ligands	Enhance absorption cross‐section and intersystem crossing leading to longer‐lived excited states. This in turn may result into higher activation yields [124].
Sterically bulky ligands at the metal centre	Destabilizes the ground state leading to easier ligand dissociation during irradiation [125].
Chelation mode (monodentate vs. polydentate ligands)	Monodentate ligands are more photolabile and easier to release compared to polydentate chelates [122].
Use of with photolabile ligands (e.g., nitriles, thiocarbonyls)	Increases sensitivity to light leading to efficient ligand dissociation [122].

As outlined in Tables 2 and 3, it can be seen that the photoactivation characteristics and therapeutic efficacy of photocaged metal complexes are greatly influenced by structural alterations. Understanding how variations in ligand types, coordination geometries and metal centres influences these characteristics have been made possible by both theoretical computations and experimental research.

**FIGURE 6:**
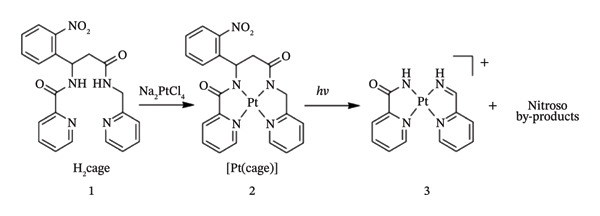
Light activation inducing bond cleavage, converting neutral [Pt(cage)] 2 into a charged and exchange labile Pt(II) complex 3. Adapted from [126].

For example, experimental work on platinum(II) photocaged complex [Pt(cage)] was described by Ciesiensk and colleagues. In their work, the photocaged complex had a photolabile nitrophenyl group integrated into the backbone of a tetradentate ligand that had two pyridyl and two amide nitrogen donor sites. As outlined in Figure 6, light activation induced bond cleavage, converting the neutral [Pt(cage)] 2 into a charged and exchange labile Pt(II) complex 3. When activated with UV light, the ligand backbone was broken, producing a Pt(II) complex that more easily exchanged its ligands with a peptide containing methionine groups. Prior to this, the intact complex was insensitive to ligand‐exchange processes [126].

In the dark, the complex had no effect on MCF‐7 breast cancer cells, however short‐term exposure to UV light caused the MCF‐7 cells to die at a rate that was comparable to cisplatin [126]. This is a new approach that might possibly deliver metal‐based medications in a site‐specific and time‐specific way by utilizing light to change the coordination chemistry surrounding the metal core.

### 9.1. Photoresponsive MPNs of Platinum

Pt‐based anticancer medicines, the first class of therapeutic anticancer metal complexes, are utilized to treat more than 40% of cancer victims because of their potent interactions with DNA nucleobases, which ultimately cause cell death. Nevertheless, their hazardous side effects and modest tumour accumulation continue to impede their clinical advancement. As a result, therapy dependent on Pt anticancer drugs is unable to produce steady therapeutic benefits.

Typically, Pt(II) complexes need UV light (*λ* < 400 nm) to activate, which has poor tissue penetration and can harm nearby tissues [127]. Although ligand loss or the oxidative production of ROS is typically involved in their photoactivation, these mechanisms are frequently less effective than in Ru(II) systems. Pt(IV) prodrugs release active Pt(II) species when activated by light, but they need to be structurally optimized for visible and NIR responsiveness.

Due to their high cytotoxicity when activated and ability to lead to the formation of DNA cross‐links, platinum complexes, particularly those based on platinum (IV), have been evaluated as a substrate for PDT [128]. Because of these qualities, they are highly effective killers of cancer cells. Nevertheless, the overwhelming toxicity of platinum‐based PSs presents highly pertinent issues associated with the biocompatibility and safety. Moreover, they are typically less photophysically favourable compared to their analogues and iridium and ruthenium complexes, especially for light harvesting and ROS generation efficiency.

Pt‐containing MPNs, on the other hand, may considerably increase therapy efficiency by enhancing the tumour accumulation impact and minimizing hazardous adverse effects to normal tissue.

Many Pt‐containing MPNs have been created for in vivo and in vitro anticancer phototherapies [8]. Since Pt(IV) azide‐based octahedral MPN compounds have lower spin d^6^ compared to the d^8^ configuration of their Pt(II) counterparts, they are less susceptible to replacement [129–131]. Additionally, Pt(IV) complexes can be rendered photoactive by selecting appropriate ligands, such as the photolabile azido group [132, 133]. Indeed, azide complexes of transition metals are often light‐sensitive. Trans‐[Pt(N_3_)_2_(CN)_4_]^2−^, which possesses a MLCT electronic absorption band (Pt ⟶ N_3_) at 302 nm, is the first photoactivated Pt(IV) complex containing azide ligands [134].

**FIGURE 7:**
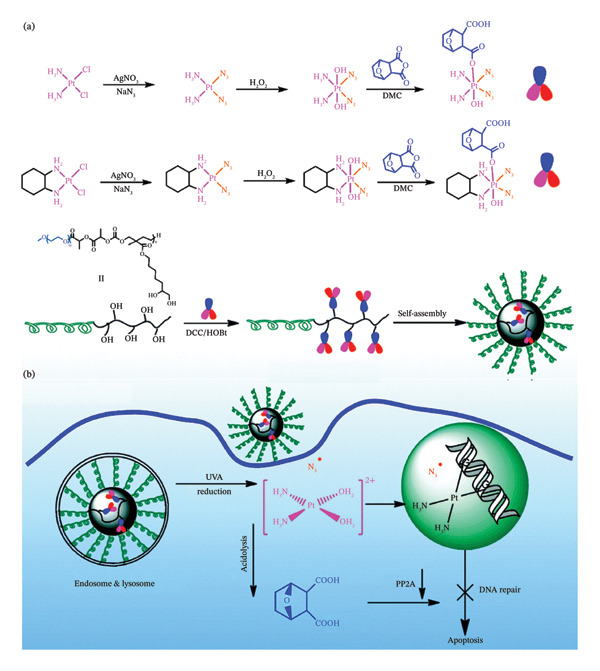
(a) Preparation of P‐Z‐DMC‐CIS(N_3_) and P‐Z‐DMC‐DXA(N_3_) micelle. (b) The working mechanisms of P‐Z‐DMC‐CIS(N_3_) micelle in cisplatin‐resistant cancer cells. Reprinted with permission from [135]. Journal of Materials Chemistry B. Royal Society of Chemistry (2015).

MPNs have been used to administer photosensitive Pt(IV)‐azide prodrugs in conjunction with other medicinal substances, in addition to their use in single‐drug photochemotherapy. There are at present two main ways that combinations are formed: either by entrapment or by linking covalent bonds to the Pt(IV)‐azide prodrugs. In order to create a multifunctional drug conjugate that can be further grafted to the side chains of copolymers, some medicines can first work in concert with Pt. Secondly, the core of MPNs can also enclose hydrophobic medicines. Chemotherapy with multidrug combinations can enhance the effectiveness of anticancer therapies by stimulating several signalling pathways in cancer cells. A photoresponsive Pt‐containing polymer (P‐Z‐DMC‐CIS(N_3_)) for multidrug chemotherapy was described by Zhou and colleagues (Figure 7(a)) [135].

The derived P‐Z‐DMC‐CIS(N_3_) had the capacity to discharge active Pt(II) and DMC inside acidic endosomes/lysosomes and was sensitive to light treatment (365 nm, 10 mW·cm^−2^). Essentially, repair of nucleotide excision and defence triggered by DNA damage requires the widely expressed protein phosphatase 2A (PP2A) (Figure 7(b)) [135]. P‐Z‐DMC‐CIS (N_3_) showed synergistic therapeutic benefits for cisplatin‐resistant cells (A549R) with a half maximum inhibitory concentration (IC50) of 8.4 μM, thanks to improved cellular absorption and photocontrolled discharge of the integrated medicines [135].

In a different instance, oxaliplatin (IV) complex and DMC were combined to create a drug conjugate, which was then grafted to the same polymer carrier to create P‐Z‐DMC‐DXA(N_3_) [136]. When used against HeLa cancer cells, the polymer nanoparticles had a remarkable synergistic effect in inhibiting cancer cell development. In addition, the P‐Z‐DMC‐DXA(N_3_) showed a positive anticancer impact, as the tumour volume progressively shrank from 100 mm^3^ to 40 mm^3^. More significantly, following P‐Z‐DMC‐DXA(N_3_) therapy and UV light irradiation (365 nm, 10 mW·cm^−2^), there was a 100% survival rate [136].

He and colleagues created a different kind of MPNs for multidrug combination treatment. Dextran‐Pt(N_3_) conjugate polymer nanoparticles (Cur@DPNs) were loaded with the photosensitive anticancer medication curcumin (Cur) (Figure 8(a)). The release of active Pt(II) and the generation of ROS were aided by the photoactivation of Cur@DPNs caused by 365 nm UVA light, which significantly reduced the viability of cancer cells (Figure 8(b)) [137]. Their results as demonstrated in Figure 8(b) demonstrate the intracellular process after endocytosis of Cur@DPNs for combinational photoactivated therapy. The overall observation is that combinational photoactivated therapy was superior to the previously reported methods of single PACT. Identifying the manner and the time frame that polyprodrug nanoparticles are photoactivated and dissociated to release active drugs in cancer cells is the primary challenge to nanoparticle‐mediated drug delivery [122].

FIGURE 8(a) Synthesis light stimulation and dual–drug sensitivity of Cur@DPNs and (b) illustration of the intracellular process after endocytosis of Cur@DPNs for combinational photoactivated therapy. Reprinted with permission from [137] Copyright, 2016. American Chemical Society.

**Figure:**
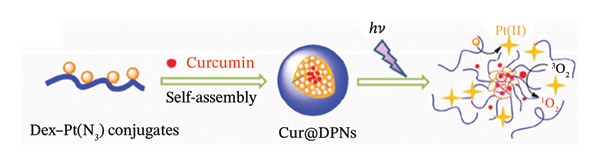
(a)

**Figure:**
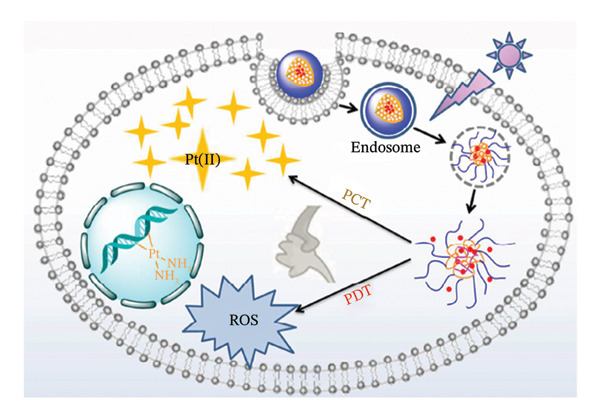
(b)

The O_2_ consumption during PDT aggravates hypoxia within a solid tumour which greatly reduces the production of ROS and further undermines the PDT efficacy. Despite one‐electron transfers from two hydroxyl groups to the Pt(IV) centre, the Pt (IV)‐azide complexes with cis‐diamine ligands could be light‐triggered to generate Pt(II) species and hydroxyl radicals that readily dimerize to hydrogen peroxide (H_2_O_2_) [138].

**FIGURE 9:**
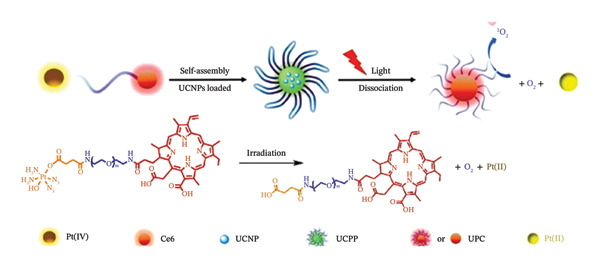
Representation of the light‐driven separation of UCPP in tumour microenvironment illustrating the formation of UCPP and its light‐driven dissociation including O_2_ production, simultaneous activation of PDT and active Pt(II) liberated for the synergistic photochemotherapy. Adapted from [139].

Under UV radiation, H_2_O_2_ then breaks down into ordinary oxygen and water. Consequently, the administration of PSs in conjunction with oxygen‐self‐generating Pt(IV) prodrug is beneficial for mitigating tumour hypoxia and promoting synergistic anticancer action. This was demonstrated by Xu and colleagues, by coupling PEG‐modified PS chlorin e6 (Ce6) to Pt(IV)‐azide complexes with cis‐diamine ligands. In order to create UCNPs‐embedded nanostructures (UCPP) with a mean size of 60 nm, the polymer was subsequently coassembled into upconversion nanoparticles (UCNPs). Figure 9 showa a representation of the light‐driven separation of UCPP in tumour microenvironment. The figure illustrates the formation of UCPP and its light‐driven dissociation including O_2_ production, simultaneous activation of PDT, and active Pt(II) liberated for the synergistic photochemotherapy [139].

Other compounds explored as NIR‐responsive candidates are Pt(IV) complexes. These compounds may break down and produce oxygen when 980 nm NIR light has been transformed by the UCNPs into shorter wavelength emissions of between 660 nm and 365 nm. The ROS self‐generating ability of UCPP remained unaltered even in a hypoxic environment under 980 nm irradiation, which reversed PDT‐induced hypoxia and increased PDT efficacy, owing to the high O_2_ level [139]. In both normoxic and hypoxic environments, L929 cells were considerably more susceptible to UCPP light exposure than the single‐function reference group. Furthermore, the fluorescence signal at the tumour location grew over the course of 24 h, and the drug’s in vivo bio distribution at several organs verified the precise build‐up of UCPP in the tumour cells [139].

Following irradiation with 980 nm laser treatment, UCPP demonstrated considerable tumour suppression efficacy with total tumour eradication in the HeLa, HCT116, and MDA‐MB‐231 tumour‐bearing animals [139]. The majority of additional haematoxylin and eosin (H&E) stain fragmentation regions and the fewest positive tumour cells detected by proliferating cell nuclear antigen (PCNA) expression were also noted. These findings showed that under NIR irradiation, UCPP may self‐generate Pt(II) and O_2_. This new photosensitive metallopolymer nanoplatform presents a promising method for mitigating hypoxia in tumours and enhancing the therapeutic impact of phototherapy.

## 10. Porphyrin‐Based MPNs of Platinum

In solid tumours, the aggressive growth of tumour cells and inadequate oxygen delivery lead to a severely hypoxic microenvironment. In this sense, the degree of hypoxia may serve as a gauge for the magnitude of the tumour. Consequently, the development of dual‐functional MPNs for combined PDT therapy and hypoxia detection is an attractive area. In this regard, Pt(II) porphyrins have garnered a lot of interest, where they are recognized for their significant stokes shift, extended triplet excited state lifetime and photostability as a typical phosphorescent transition‐metal complex (PTMC).

Zhou and coworkers conducted a ground‐breaking study wherein they utilized Suzuki coupling polymerization to include Pt(II) porphyrin into a hyperbranched linked polyelectrolyte that was initially insensitive to oxygen (Figure 10(a)) [140]. The polyelectrolytes could build up in tumour cells through the EPR effect as they self‐assembled into polymer dots (Pdots) with a mean size of around 10 nm. Pdots’ ^1^O_2_ quantum yield reached 0.80 (*λ*_ex_ = 532 nm) as a result of intramolecular Forster resonance energy transfer (FRET) increasing light absorption from the conjugated backbone to the acceptor Pt(II) porphyrin [141]. In addition, the polymer’s three‐dimensional structure helped to lessen self‐aggregation and undesirable intermolecular π‐π stacking. Furthermore, ratiometric O_2_ recognition was made possible by the FRET from polyfluorene units to Pt(II) porphyrin [141].

FIGURE 10(a) Chemical structure, TEM image of polymer dots (Pdots) in PBS solution and O_2_‐sensing mechanisms during PDT of the Pt(II) porphyrin‐containing Pdots [140]. (b) Chemical structures of Por(Pt)‐bisUPy and DPA‐bisUPy. Reprinted with permission from [141]. Materials Chemistry Frontiers. The Royal Society of Chemistry (2018).

**Figure:**
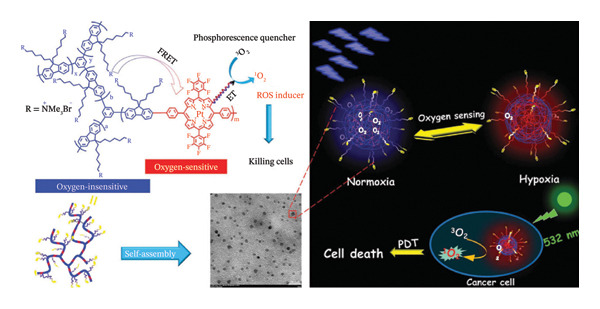
(a)

**Figure:**
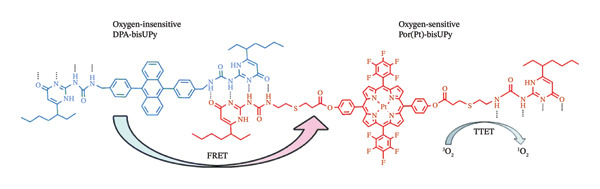
(b)

Analysis of apoptosis and necrosis in HepG2 cells revealed that polymer dots had minimal dark cytotoxicity and that the ROS they produced was what caused the cell mortality to accelerate as the exposure period increased [142, 143]. This shows that PDT therapies and intracellular hypoxia recognition are feasible at the same time, and it offers vital guidance for the creation of phosphorescent diagnosis‐therapy integrated systems in the future [140].

Huang et al. assembled a quadruple hydrogen‐bonded supramolecular polymer to create another bifunctional Pt(II) porphyrin MPN for ratiometric hypoxia detection and PDT. Their procedure is illustrated in Figure 10(b), which shows the chemical structure, TEM image of polymer dots (Pdots) in PBS solution and O_2_ sensing mechanisms during PDT of the Pt(II) porphyrin‐containing Pdots [141]. The oxygen‐insensitive fluorescence reference moiety carrying bis‐UPy served as the chemotherapeutic energy absorber in this approach, while the oxygen‐sensitive Pt(II) porphyrin group holding ureidopyrimidinone (bis‐UPy) served as the energy source. The MPNs have a straightforward synthesis and are biodegradable due to the noncovalent linkage between the monomers. The uniformly tiny particle size of the MPNs (96 nm) allowed them to easily disperse in aqueous solutions and enter tumour cells. The ^1^O_2_ quantum yield (*λ*_ex_ = 532 nm) was found to be 0.70, indicating that MPNs have a significant anticancer therapeutic potency [141].

A summary of some of these complexes in various experimental stages is tabulated in Table 4. These outline the progress in research for these compounds with the mechanisms explored.

**TABLE 4:** Examples of complexes in various stages from laboratory to clinical trials.

Compound	Mechanism	Stage	References
TLD‐1433	ROS production, PDT	Clinical trials (bladder cancer)	[144, 145]
Pt(IV)‐DACH prodrugs	Pt(II) release, DNA binding	Preclinical	[146]
[Ru(bpy)_2_(L)]^2+^ with caged HDAC inhibitors	Enzyme inhibition	Lab studies	[147]
Pt(IV)‐diazido complexes	ROS + Pt(II) generation	Preclinical	[148]
[Ru(bpy)_2_(L‐drug)]^2+^ photocages	A cytotoxic small molecule (such as an analogue of nicotinamide or a NAMPT inhibitor) is released when exposed to light; this results in cytotoxicity.	Preclinical – cell and animal studies only. No human trials to date.	[149]
Ru‐caged microtubule‐inhibitor (red‐light activatable)	Microtubule polymerization inhibitor induced by light; shown in vitro and in vivo models	Preclinical (recent reports, in vivo tumour models).	[17]
Ru‐STF31/Ru‐linked metabolic inhibitors	NAD^+^ depletion and metabolic disruption in cancer cells are caused by the release of metabolic/targeted inhibitors (such as STF31 derivatives).	Preclinical (cell experiments reported; early animal data in some studies).	[150]
Ru(II) photocages for enzyme inhibitors/CYP inhibitors (Havrylyuk et al.)	Accurate regulation of enzyme inhibitors (proof‐of‐concept photocages that, when exposed to light, release active inhibitors)	Preclinical/chemical‐biology tools	[151]
Dual‐action Ru complexes (PACT + PDT) (recent designs)	To increase effectiveness and get over hypoxic restrictions, combine a photo‐released medication with photosensitization.	Preclinical (concept and animal data reported)	[152]
Ru‐DOX/polymeric Ru precursors for DOX photorelease	Chemotherapeutic release driven by light or photo‐enhanced antimicrobial activity (proof of concept)	Preclinical	[153]

## 11. Organic Photoswitches and PPGs

PPGs were first employed in experiments for biological research in the 1970s [154]. As illustrated in Scheme 5, PPGs are designed with the intention of masking a substrate’s bioactive moieties by covalently attaching the protective group to a molecule that is essential to the action (a process known as “caging”) [155]. These PPGs are some of the most exploited photoswitches in biological applications. They undergo E/Z photoisomerization and photocyclization/ring opening.

SCHEME 5Overview of the most exploited photoswitches in biological applications. (a) E/Z photoisomerization‐based molecular photoswitches. (b) Photocyclization/ring opening‐based molecular switches. Adapted from [155] Doctoral dissertation, Universidad de la Rioja, 2021.

**Figure:**
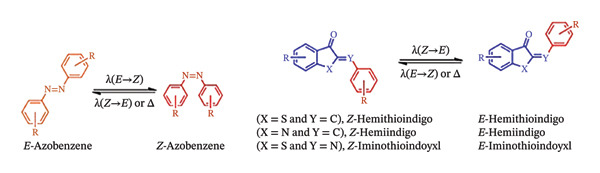
(a)

**Figure:**
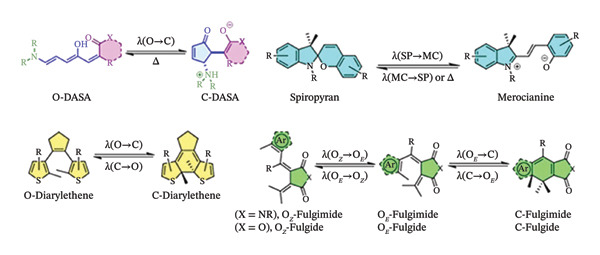
(b)

By eliminating the PPG with light irradiation – that is, by converting the absorbed energy into a photocleavage process known as “un‐caging” – the activity may be reinstated on demand and in a spatiotemporally controlled way [44]. In this instance, the photoactivation is irreversible, which is frequently viewed as a drawback of the methodology [156]. Even if a stoichiometric quantity of the PPG would also be present in the medium, there is still a chance that the released active agent(s) would have undesirable effects upon diffusion or excretion, generating further safety concerns [157, 158].

The research area of light‐responsive (small) drug molecules faces many of the same difficulties as other drug delivery systems, such as tissue permeation, transportation to and accumulation at the active site, control over activation signal in vivo, materials and instrumentation complexity, available translational models, and safety.

**SCHEME 6:**
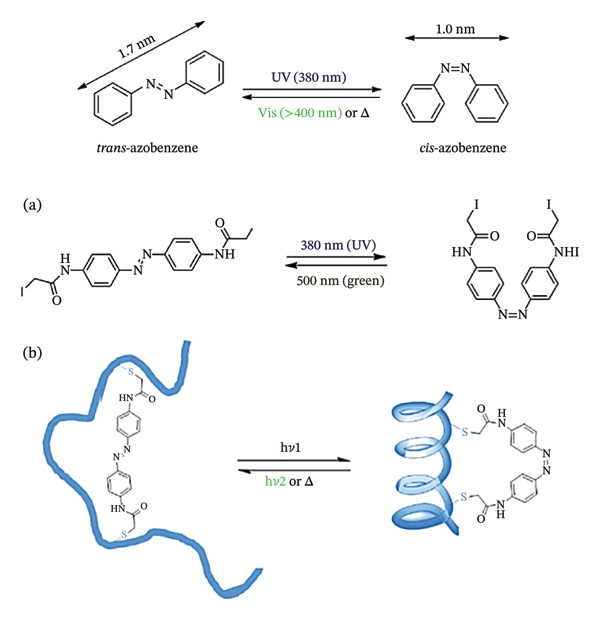
Structures of *trans-* and *cis*‐azobenzene. (a) Chemical structure of the diiodoacetamide azobenzene photoswitch, with electrophilic groups at two ends for covalent linkage to peptides. (b) Schematic diagram for the control of the peptide structure upon isomerization of the photoswitch. Reprinted with permission from [162]. Organic and Biomolecular Chemistry, Royal Society of Chemistry, 2018.

The frequency of the light needed for tissue penetration, and consequently, photoactivation, presents a unique challenge. Longer wavelength absorption, for example, often results in quicker thermal relaxation in the field of azobenzene photoswitches, a family of chemical compounds that, when exposed to light, easily isomerize between different geometries as shown in (Scheme 6) [159–162]. Moreover, the relative stabilities of the Z and E isomers may also change.

This makes probes that operate at higher wavelengths (red, infrared) or, preferably, in the biological window (*λ* = 650–1450 nm) advantageous, and a lot of work has gone into creating photoactivatable units in these longer wavelength ranges. A common strategy to move the absorption to longer wavelengths is to set up push–pull systems with EDG and electron‐withdrawing group arranged strategically.

In addition to chromophore modifications, certain formulations may facilitate the utilization of longer wavelength light. For example, employing UCNPs or different light sources as the Cherenkov radiation (i.e., a design predicated on internal co‐localization of the light source and the photoactive agent) could be one such formulation. Currently, a significant obstacle is that most papers discussing photoactivatable probes rely on in vitro tests (proof‐of‐concept research). These shows how applicable the idea is in general. Gaining more understanding of how these systems could function in vivo would be crucial for potential therapeutic uses. Restoring eyesight is one of the most researched areas for in vivo use of photoactivatable drugs to date [163].

## 12. Organic Versus Metal Photocages

Although organic compounds have long been used as photocages, they do have some significant disadvantages. Many organic photocaging units need non‐bioorthogonal UV light in order to generate photorelease [81]. On the other hand, metal photocages have the ability to release nitriles and aromatic heterocycles, which are known to be significant moieties in heme‐ and protein‐binding interactions. They also exhibit the capacity to go through electronic transitions required for uncaging at longer wavelengths [164]. This is made possible by the weaker bonding interactions between metal centres and their ligands (compared to the stronger organic σ‐bonds). Overall, these properties in metal photocages contributing to greater ease of photodissociation provide advantages over the traditional organic photocages.

Moreover, metal complexes provide the fascinating possibility of easily adjusting the overall photophysical characteristics of the complex by modifying the structures of the auxiliary ligands. By adding steric bulk to the bipyridyl ligand, for instance, [Ru(tpy) (bpy) (py)]^2+^ can be transformed into an effective photocage [Ru(tpy) (Me2bpy) (py)]^2+^ [164, 165]. This tactic is known to upset the coordination complex’s octahedral geometry and stabilize its 3MC state, which facilitates the complex’s transition from the 3MLCT into the dissociative 3MC state. Therefore, adding steric bulk to auxiliary ligands of a metal‐centred complex is a commonly used method to create effective and dependable Ru(II) photocages.

### 12.1. Synergistic Effects From Photoactivatable Inorganic Complexes With Immunotherapy and Gene Therapy

Due to tumour heterogeneity and adverse treatment resistance, single formulations seldom achieve the intended therapeutic effects in clinical trials [166]. There has been an increasing interest in synergistic combination treatments, which combine two or more therapeutic modalities. Gene therapy is a multipurpose treatment for cancer that can induce apoptosis in cancer cells, prevent carcinogenesis or metastasis, downregulate heat shock proteins, and stimulate the immune system by producing cytokines [167].

A possible method for improving the effectiveness of cancer treatment is the combination of immunotherapy, gene therapy, and photoactivatable inorganic compounds [168]. This strategy makes use of the systemic advantages of gene and immunotherapies as well as the exact control of phototherapy to provide synergistic effects on malignancies. By using multifunctional modes of action, the objective is to increase selectivity, decrease adverse effects, and maximize synergistic benefits [169]. Tumour‐associated antigens (TAAs) and danger‐associated molecular patterns (DAMPs), which excite dendritic cells and activate cytotoxic T lymphocytes, are released when phototherapy induces Immunogenic Cell Death (ICD) [170]. The body’s immunological reaction against malignancies is strengthened by this process.

Due to its superiority in therapeutic enhancement and reducing adverse effects, gene therapy is currently a useful adjunct to PTT/PDT [171]. There are two primary methods via which gene therapy and PTT work in concert. Either gene therapy and PTT work together to kill tumour cells, allowing gene therapy to kill nearby or PTT‐resistant tumour cells, or gene therapy can increase tumour cells’ susceptibility to PTT by, for example, preventing the production of heat shock proteins, which increases PTT’s effectiveness [171]. Gold nanoparticles (GNs), carbon‐based materials, black phosphorus nanosheets (BP NS) and other inorganic materials have all been used in gene/photothermal combination treatment [172]. Certain inorganic nanomaterials must be modified in order to load therapeutic nucleic acids; for example, the surface of the material may need to be modified with a cationic polymer to load the nucleic acid by electrostatic contact with the nucleic acid [173].

## 13. Recent Advances in Photocaged Compounds

Recent advances in this field have proved it to be a promising area in cancer therapy. Most notable research has been carried out by Guangyu Zhu and coworkers, who have come up with ground breaking findings in the applications of light‐activable compounds in disease treatment. They worked on customized precision medicines in cancer treatment with distinct mechanisms of action for overcoming drug resistance. Here, they came up with targeted anticancer prodrugs with controlled activation. Their prodrug named coumaplatin was a photocaged Pt(IV) prodrug based on oxaliplatin that achieved both nuclear accumulation and “on‐demand” activation as illustrated in Figure 11 [174]. The Pt(IV) complex on which this prodrug was based was found to be effectively photoactivated by water oxidation without the need of a reducing agent [174]. The complex was found to be stable in the dark but effectively activated upon irradiation with low‐intensity visible light.

FIGURE 11(a) Coumarin and (b) the proposed photoactivation mechanism of a coumarin analogue (Complex 3) in PBS buffer (pH 7.4). Reprinted with permission from [174].

**Figure:**
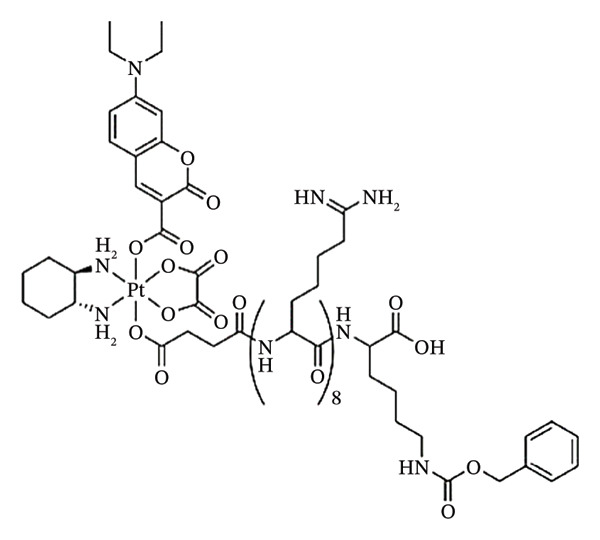
(a)

**Figure:**
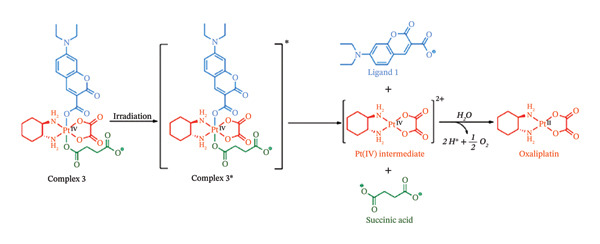
(b)

When the prodrug was photoactivated, it showed a degree of photocytotoxicity which was up to two orders of magnitude greater than that of oxaliplatin, and was also found to accumulate extremely quickly in the nucleoli [174]. Surprisingly, this prodrug had a markedly improved capacity for tumour penetration and employed a unique mechanism of action to overcome drug resistance and cause immunogenic cell death, p53‐independent cell death and cell senescence in addition to T cell activation [174].

Their results not only offer a new approach to the rational drug design of nucleolus‐targeted and controllably activated Pt(IV) anticancer prodrugs but also show that delivering traditional platinum medications to the nucleus is a workable method of altering their canonical mechanism of action and reduce resistance.

## 14. Challenges and Shortcomings With Photoactivation

Despite the prospects held by photoactivation approach in cancer therapy, the development and application of photocaged medications continue to present a number of challenges. Most of the time, challenges in solubility require the usage of other molecules, including nanocarriers, to make them useful in therapy [175, 176]. Dark toxicity, or toxicity in the absence of light, is something that the perfect PS should not have. The ideal illumination should cause little off‐target retention and accumulation while causing targeted harm to every part of the tumour, including its vasculature. Strong antitumour immune responses that can eradicate distant metastatic lesions and residual tumour cells are characteristics of the ideal PS. Once the anticancer effect has been demonstrated, a favourable biodistribution should promote little to no off‐target effects, little or very little skin photosensitivity, and quick body clearance. These challenges to a large extent still remain active areas of concern and research in the application of photocages.

Photoactivation is an oxygen‐dependent process, which is a challenge in hypoxic sites. Furthermore, the PS utilized needs to show specificity for sites of neoplasia, and be activated by light at wavelengths that can readily penetrate into tissues. The greatest tissue penetration depth of the light employed for therapy, which is reliant on the attenuation and propagation of light in tissues, presents another difficulty for PDT treatment [177, 178]. The processes of reflectance, absorption, scattering and refraction are responsible for this reduction of light passing through tissues. The most significant factor affecting both the directionality and intensity of light attenuation in tissues, such as tumours, is the scattering of light inside such tissues. The absorption spectra of the chromophores found in tissues, including water, oxy‐ and deoxyhemoglobin, melanin and different cytochromes, establish the ideal spectrum in terms of light penetration. Absorption of light also has a significant influence on the decrease in light intensity. All of these factors which affect how light moves through different media have a major impact on the depth of penetration in tissues, and this in turn affects how well photoactivable devices can be utilised for treatment.

The “drug‐to‐light interval” (DLI) is another issue that arises while using PDT therapy [179–181]. For PDT to produce a photodynamic effect, illumination from a light source is necessary. This is carried out after the PDT medication has been administered to the body. The DLI is the amount of time that passes between the PDT drug’s administration and illumination at the appropriate wavelength. The DLI for PDT is frequently selected to cause damage to the regions of interest; notably, the tumour, the vasculature or a combination of both is dependent on the pharmacokinetics of the PS. If PS is administered intravenously and then spread to the tumour, a brief DLI would primarily target the vasculature. Given that it would have enough time to build up inside the tumour, a long DLI would primarily target the tumour cells, whereas a medium DLI would target both [182].

The tumour size may surpass the maximal penetration depth of light, making PDT less effective even when employing light inside the therapeutic window [93, 177, 183]. Consequently, the tumours that can be treated with PDT depend on the greatest propagation of the treatment light and the sites that can be reached by a light source. Because of this, PDT is frequently used to treat tumour types such as surface malignancies or conditions that may be reached with a flexible light source. These cancers include those of the bile duct, bladder, liver, colon, pancreas, brain, ovarian and prostate cancers, in addition to a number of cutaneous malignancies, such as basal cell carcinoma and squamous cell carcinoma, head and neck, esophageal and lung cancers.

The other major challenge in PDT is hypoxia [184]. The hypoxic environment that solid tumours frequently exhibit can provide a challenge to PDT’s effectiveness, since the principal acceptor of light energy, oxygen, is scarce at the site of action. This can lessen the therapeutic impact by reducing photodynamic damage through singlet oxygen production. PSs, which does not need a lot of oxygen to operate, are being used in recent attempts to alleviate hypoxia in the context of PDT [172].

## 15. Summary

In summary, this report discusses in depth the principles and mechanisms of both PDT and photocaging and photouncaging processes. These have been explained by the use of relevant figures and schemes for clarity. The factors that make photocaging route of drug delivery in cancer are well discussed from a background of literature reports which report laboratory scale in vitro and in vivo tests. Examples of these compounds are highlighted together with the opportunities for future research in this interesting field. Selective accumulation at sites of neoplasia (targeting) and the penetration of light as critical factors have both been discussed in the manuscript. The various biological targets and the mode of action have also been discussed. The challenges and opportunities appear in the various sections of the manuscript, with the areas of concern highlighted in the ‘challenges’ section.

## 16. Conclusion: Way Forward and Future Clinical Outlook

In conclusion, possible future reach and potential of light‐activatable compounds may provide real‐time drug release surveillance (i.e., real‐time radiation exposure data). For photocages, the basic method for real‐time photorelease measurement is the release or creation of a fluorescent species. Obtaining quantitative data on free drug concentration is extremely useful, especially as we move toward in vivo systems. Light‐triggered action might potentially be enhanced by imaging modalities, especially in the context of nanodevices [104].

Notwithstanding these benefits, a number of drawbacks exist when it comes to the production of these compounds. Iridium (III) complexes, for example, can be expensive and difficult to synthesize, which may prevent widespread use in medicine and large‐scale manufacturing [185]. In order to completely comprehend the long‐term effects and safety profile of these complexes, extensive in vivo toxicity studies are still necessary and pending. Furthermore, even if NIR activation enhances tissue penetration, PDT’s efficacy is still constrained by the depth of light penetration challenges, which makes it less appropriate for tumours that are deeply lodged into tissues [186].

In order to improve the bioavailability and targeting of iridium (III) complexes, future research should concentrate on creating delivery methods which impart selective distribution and regulated release of these PSs to tumour locations. These might be improved by innovations like stimuli‐responsive carriers and multifunctional nanoparticles. Examining combination treatments that combine PDT with other therapeutic approaches may improve overall therapeutic results. For example, PDT and immune checkpoint inhibitors may work in concert to enhance antitumour immune response [187]. Clinical trials and long‐term in vivo research are necessary to thoroughly assess the safety, effectiveness and any adverse effects of these complexes.

An interesting area for further study is the use of these complexes in the treatment of ailments other than cancer, such as bacterial infections and other proliferative disorders. New treatment approaches for a wider variety of medical applications might be developed through drug repurposing, by utilizing their already understood properties. In summary, although the therapeutic application of these complexes in cancer treatment shows promise, it requires the creation of effective drug delivery methods, thorough toxicity analyses and clinical protocol models, in order to successfully incorporate these complexes into clinical practice and perhaps enhance the results of cancer treatment. It is however encouraging to know that their future clinical research and the potential clinical use in cancer therapy are made possible by the ongoing developments in this sector, which are backed by strong preclinical findings.

Overall, finding innovative APIs or anticancer drug candidates remains an extremely difficult endeavour. Regulatory difficulties, distinct pharmacokinetic features of the isomers and the reliance of the photochemical properties on the physiological environment are only a few of the additional challenges that arise when one layer of complexity is added. Except for a limited number of in vivo animal experiments, cancer cell line tests are the primary means of validating biological activity at the current stage of light‐responsive anticancer medicines, which are centred on improving chemical characteristics. Due to the continual improvement of structures and rapid developments, research is moving quickly toward evaluating photoresponsive chemicals in animal models in order to validate projected advancements in cancer treatment.

Based on the foregoing discussions, the future of these photoactivable complexes will go from proof‐of‐concept PDT to a clinically feasible platform for spatiotemporally precise chemotherapy. The drawbacks, majorly on the inherent challenges of light penetration, tumour hypoxia and immune recognition, will have to be overcome by achieving localized drug activation with low systemic toxicity.

The creation of prodrugs and PSs that respond to NIR light (700–1000 nm) will be of paramount importance in the near future considering its deeper tissue accessibility, which allows for less invasive diagnosis and treatment of internal cancers. This could be made possible by coupling PSs with UCNPs, which transform NIR to visible or UV light while minimizing radiation damage to normal tissue. Recent studies have focussed on advancements in UCNP fabrication, efficiency in conversion and biological compatibility which could render this approach translationally feasible over the next 10 years.

Due to its reliance on singlet‐oxygen production, conventional PDT is constrained by tumour hypoxia. For hypoxic microenvironments, Type I PSs and PACT systems that produce radicals or superoxide by electron transfer processes are becoming attractive alternatives. Redox‐tunable chromophores and BODIPY/aza‐BODIPY composites with logical designs will increase their effectiveness in hypoxic lesions.

X‐ray or Cerenkov‐mediated PDT (X‐PDT/CR‐PDT) is another cutting‐edge method for inaccessible tumours that uses radiation technology to initiate photochemical processes deep inside tissues. At the same time, theranostic drugs that combine activation capabilities with imaging modalities like MRI, PET and fluorescence will enable real‐time therapy monitoring and planning, which is essential for clinical application. These procedures chart a viable near future application for these promising photocaged compounds.

Crosscutting support and concerted collaborations involving chemists, oncologists, immunologists and medical engineers will be necessary to realize these advancements. Within the next five to 10 years, the field of photocaged therapeutics can produce NIR‐responsive, image‐guided, immunogenic photochemotherapeutic systems. If these strategies are carefully pursued, they will be able to clinically address challenges with deep‐tissue access, with targeted tumour control.

## Author Contributions

All the authors contributed similar efforts during the preparation of the manuscript.

## Funding

No funding was received for this manuscript.

## Conflicts of Interest

The authors declare no conflicts of interest.

## References

[1] Zhao Y. , Jiang X. , Liu X. , Liu X. , Liu Z. , and Liu X. , Application of Photo-Responsive Metal-Organic Framework in Cancer Therapy and Bioimaging, Frontiers in Bioengineering and Biotechnology. (2022) 10, 10.3389/fbioe.2022.1031986. PMC9633982 36338113

[2] Imran M. , Ayub W. , Butler I. S. , and Zia-ur-Rehman , Photoactivated Platinum-Based Anticancer Drugs, Coordination Chemistry Reviews. (2018) 376, 405–429, 10.1016/j.ccr.2018.08.009, 2-s2.0-85052432665.

[3] Yu Y. , Xu Q. , He S. et al., Recent Advances in Delivery of Photosensitive Metal-Based Drugs, Coordination Chemistry Reviews. (2019) 387, 154–179, 10.1016/j.ccr.2019.01.020, 2-s2.0-85061790120.

[4] Kansız S. and Elçin Y. M. , Advanced Liposome and Polymersome-Based Drug Delivery Systems: Considerations for Physicochemical Properties, Targeting Strategies and Stimuli-Sensitive Approaches, Advances in Colloid and Interface Science. (2023) 317, 10.1016/j.cis.2023.102930. 37290380

[5] Rezaei A. , Rafieian F. , Akbari-Alavijeh S. , Kharazmi M. S. , and Jafari S. M. , Release of Bioactive Compounds From Delivery Systems by Stimuli-Responsive Approaches; Triggering Factors, Mechanisms, and Applications, Advances in Colloid and Interface Science. (2022) 307, 10.1016/j.cis.2022.102728. 35843031

[6] Smith N. A. and Sadler P. J. , Photoactivatable Metal Complexes: from Theory to Applications in Biotechnology and Medicine, Philosophical Transactions of the Royal Society A: Mathematical, Physical and Engineering Sciences. (2013) 371, no. 1995, 10.1098/rsta.2012.0519, 2-s2.0-84887352833. PMC3685452 23776303

[7] Madec H. , Figueiredo F. , Cariou K. , Roland S. , Sollogoub M. , and Gasser G. , Metal Complexes for Catalytic and Photocatalytic Reactions in Living Cells and Organisms, Chemical Science. (2023) 14, no. 3, 409–442, 10.1039/d2sc05672k. 36741514 PMC9848159

[8] He M. , Chen F. , Shao D. , Weis P. , Wei Z. , and Sun W. , Photoresponsive Metallopolymer Nanoparticles for Cancer Theranostics, Biomaterials. (2021) 275, 10.1016/j.biomaterials.2021.120915. 34102525

[9] Ai X. , Hu M. , and Xing B. , Photoactivatable Targeting Methods, Handbook of In Vivo Chemistry in Mice: From Lab to Living System. (2020) Wiley, Hoboken, NJ, 401–432.

[10] Ng C. H. , Kim S. L. , Kevlishvili I. et al., Visible-Light-Mediated Macrocyclization for the Formation of Azetine-Based Dimers, ACS Catalysis. (2024) 14.

[11] Song S. , Lee D. , Dalle Ore L. C. et al., Photoactivable Liposomes for Controlled Delivery: Recent Progress and Design Considerations, Coordination Chemistry Reviews. (2024) 501, 10.1016/j.ccr.2023.215567.

[12] Gold M. H. , Photodynamic Therapy: Indications and Treatment, Aesthetic Surgery Journal. (2008) 28, no. 5, 545–552, 10.1016/j.asj.2008.08.007, 2-s2.0-53449085061. 19083578

[13] Dunkel P. and Ilaš J. , Targeted Cancer Therapy Using Compounds Activated by Light, Cancers (Basel). (2021) 13, no. 13, 10.3390/cancers13133237. PMC8269035 34209493

[14] Josa‐Culleré L. and Llebaria A. , In the Search for Photocages Cleavable With Visible Light: An Overview of Recent Advances and Chemical Strategies, ChemPhotoChem. (2021) 5, no. 4, 296–314, 10.1002/cptc.202000253.

[15] Liu L. , Zhang D. , Johnson M. , and Devaraj N. K. , Light-Activated Tetrazines Enable Precision Live-Cell Bioorthogonal Chemistry, Nature Chemistry. (2022) 14, no. 9, 1078–1085, 10.1038/s41557-022-00963-8. PMC10198265 35788560

[16] Zhu W. F. , Empel C. , Pelliccia S. , Koenigs R. M. , Proschak E. , and Hernandez-Olmos V. , Photochemistry in Medicinal Chemistry and Chemical Biology, Journal of Medicinal Chemistry. (2024) 67. 10.1021/acs.jmedchem.3c02109 38457829

[17] Bretin L. , Husiev Y. , Ramu V. et al., Red‐Light Activation of a Microtubule Polymerization Inhibitor via Amide Functionalization of the Ruthenium Photocage, Angewandte Chemie International. (2024) 63, no. 5, 10.1002/ange.202316425. 38061013

[18] Santosh S. , Rajagopalan M. D. , Pallavi B. A. et al., Cancer Therapies: Current Scenario, Management, and Safety Aspects, Anticancer Plants: Clinical Trials and Nanotechnology: Volume 3. (2017) Springer, Berlin, 1–25.

[19] Singh B. and Gaitonde R. U. , Application of Two-Dimensional Materials for Cancer Theranostic, Nanomaterials in Healthcare, 2024, CRC Press, Boca Raton, FL, 259–279.

[20] Taylor R. A. , Jalali M. , and Marti J. , Nanotechnology: Creating, Manipulating, and Observing Nanostructured Systems in Biology and Medicine, Pharmacognosy, 2024, Elsevier, Amsterdam, 727–747.

[21] Karewicz A. , Lachowicz D. , and Pietraszek A. , Photonics in Drug Delivery, Polymer and Photonic Materials Towards Biomedical Breakthroughs. (2018) Springer, Berlin, 131–151.

[22] Bown S. G. , Photodynamic Therapy for Photochemists, Philosophical Transactions of the Royal Society A: Mathematical, Physical and Engineering Sciences. (2013) 371, no. 1995, 10.1098/rsta.2012.0371, 2-s2.0-84887333490. 23776302

[23] Yao Q. , Fan J. , Long S. et al., The Concept and Examples of Type-III Photosensitizers for Cancer Photodynamic Therapy, Chem. (2022) 8, no. 1, 197–209, 10.1016/j.chempr.2021.10.006.

[24] Correia J. H. , Rodrigues J. A. , Pimenta S. , Dong T. , and Yang Z. , Photodynamic Therapy Review: Principles, Photosensitizers, Applications, and Future Directions, Pharmaceutics. (2021) 13, no. 9, 10.3390/pharmaceutics13091332. PMC8470722 34575408

[25] Leonidova A. , Pierroz V. , Rubbiani R. et al., Photo-Induced Uncaging of a Specific Re (I) Organometallic Complex in Living Cells, Chemical Science. (2014) 5, no. 10, 4044–4056, 10.1039/c3sc53550a, 2-s2.0-84906699461.

[26] Kuzmina N. S. , Otvagin V. F. , Maleev A. A. et al., Development of Novel Porphyrin/Combretastatin A-4 Conjugates for Bimodal Chemo and Photodynamic Therapy: Synthesis, Photophysical and TDDFT Computational Studies, Journal of Photochemistry and Photobiology A: Chemistry. (2022) 433, 10.1016/j.jphotochem.2022.114138.

[27] Shymborska Y. , Budkowski A. , Raczkowska J. et al., Switching It Up: The Promise of Stimuli‐Responsive Polymer Systems in Biomedical Science, Chemical Record. (2024) 24, no. 2, 10.1002/tcr.202300217. 37668274

[28] Buruiana L. I. , Multifunctional Materials for Biotechnology: Opportunities and Challenges, Industrial Applications for Intelligent Polymers and Coatings. (2016) Springer, Berlin, 337–353.

[29] Mena-Giraldo P. and Orozco J. , Polymeric Micro/Nanocarriers and Motors for Cargo Transport and Phototriggered Delivery, Polymers. (2021) 13, no. 22, 10.3390/polym13223920. PMC8621231 34833219

[30] Tan X. , Li B. B. , Lu X. et al., Light-Triggered, Self-Immolative Nucleic Acid-Drug Nanostructures, Journal of the American Chemical Society. (2015) 137, no. 19, 6112–6115, 10.1021/jacs.5b00795, 2-s2.0-84930225617. 25924099

[31] Rylands M. , Syntheses of Luciferins and Their Bioluminescent Evaluation, 2018, University of Cape Town.

[32] McCoy C. P. , Rooney C. , Edwards C. R. , Jones D. S. , and Gorman S. P. , Light-Triggered Molecule-Scale Drug Dosing Devices, Journal of the American Chemical Society. (2007) 129, no. 31, 9572–9573, 10.1021/ja073053q, 2-s2.0-34547776440. 17636919

[33] Tavakkoli Yaraki M. , Liu B. , and Tan Y. N. , Emerging Strategies in Enhancing Singlet Oxygen Generation of Nano-Photosensitizers Toward Advanced Phototherapy, Nano-Micro Letters. (2022) 14, no. 1, 10.1007/s40820-022-00856-y. PMC9072609 35513555

[34] Pirrung M. C. and Rana V. S. , Photoremovable Protecting Groups in DNA Synthesis and Microarray Fabrication, Dynamic Studies in Biology: Phototriggers, Photoswitches, and Caged Biomolecules. (2006) John Wiley Sons, Hoboken, NJ.

[35] Wei Y. , Yan Y. , Pei D. , and Gong B. , A Photoactivated Prodrug, Bioorganic & Medicinal Chemistry Letters. (1998) 8, no. 18, 2419–2422, 10.1016/s0960-894x(98)00437-5, 2-s2.0-17044442290. 9873553

[36] Aleem S. and Ryan U. , BODIPY-Derived Photoremovable Protecting Groups Unmasked With Green Light, 2015. 10.1021/jacs.5b01297 25751156

[37] Nguyen V. , Yim Y. , Kim S. et al., Molecular Design of Highly Efficient Heavy‐Atom‐Free Triplet BODIPY Derivatives for Photodynamic Therapy and Bioimaging, Angewandte Chemie International Edition. (2020) 59, no. 23, 8957–8962, 10.1002/anie.202002843. 32125064

[38] Adhikari S. , Moscatelli J. , Smith E. M. , Banerjee C. , and Puchner E. M. , Single-Molecule Localization Microscopy and Tracking With Red-Shifted States of Conventional BODIPY Conjugates in Living Cells, Nature Communications. (2019) 10, no. 1, 10.1038/s41467-019-11384-6, 2-s2.0-85069975092. PMC6667493 31363088

[39] Goswami P. P. , Syed A. , Beck C. L. et al., BODIPY-Derived Photoremovable Protecting Groups Unmasked With Green Light, Journal of the American Chemical Society. (2015) 137, no. 11, 3783–3786, 10.1021/jacs.5b01297, 2-s2.0-84926195512. 25751156

[40] Kand D. , Liu P. , Navarro M. X. et al., Water-Soluble BODIPY Photocages With Tunable Cellular Localization, Journal of the American Chemical Society. (2020) 142, no. 11, 4970–4974, 10.1021/jacs.9b13219. 32115942 PMC7302507

[41] Xiong H. , Xu Y. , Kim B. et al., Photo-Controllable Biochemistry: Exploiting the Photocages in Phototherapeutic Window, Chem. (2023) 9, no. 1, 29–64, 10.1016/j.chempr.2022.11.007.

[42] Bandl C. , Kern W. , and Schlögl S. , Adhesives for “Debonding-on-Demand”: Triggered Release Mechanisms and Typical Applications, International Journal of Adhesion and Adhesives. (2020) 99, 10.1016/j.ijadhadh.2020.102585.

[43] Lee H.-M. , Larson D. R. , and Lawrence D. S. , Illuminating the Chemistry of Life: Design, Synthesis, and Applications of “Caged” and Related Photoresponsive Compounds, ACS Chemical Biology. (2009) 4, no. 6, 409–427, 10.1021/cb900036s, 2-s2.0-67649243556. 19298086 PMC2700207

[44] Li Y. , Wang M. , Wang F. , Lu S. , and Chen X. , Recent Progress in Studies of Photocages, Smart Molecules. (2023) 1, no. 1, 10.1002/smo.20220003. PMC12118285 40625648

[45] Izumi Y. , Ohara M. , Fujii K. , Yokoya A. , and Ogawa M. , X-Ray Photoemission and Absorption Spectroscopy of a Hypervalent Iodine Compound, 2-Iodosobenzoic Acid, Nuclear Instruments and Methods in Physics Research Section B: Beam Interactions With Materials and Atoms. (2024) 547, 10.1016/j.nimb.2023.165211.

[46] Martínez-Alonso M. , Jones C. G. , Shipp J. D. , Chekulaev D. , Bryant H. E. , and Weinstein J. A. , Phototoxicity of Cyclometallated Ir (III) Complexes Bearing a Thio-Bis-Benzimidazole Ligand, and Its Monodentate Analogue, as Potential PDT Photosensitisers in Cancer Cell Killing, Journal of Biological Inorganic Chemistry. (2024) 29, 1–13, 10.1007/s00775-023-02031-z. 38183420 PMC11001735

[47] Lowry M. S. and Bernhard S. , Synthetically Tailored Excited States: Phosphorescent, Cyclometalated Iridium (III) Complexes and Their Applications, Chemistry – A European Journal. (2006) 12, no. 31, 7970–7977, 10.1002/chem.200600618. 16933348

[48] Lalitha R. and Velmathi S. , A Study of Small Molecule-Based Rhodamine-Derived Chemosensors and Their Implications in Environmental and Biological Systems From 2012 to 2021: Latest Advancement and Future Prospects, Journal of Fluorescence. (2024) 34, no. 1, 15–118, 10.1007/s10895-023-03231-1. 37212978

[49] Wegeberg C. , Häussinger D. , Kupfer S. , and Wenger O. S. , Controlling the Photophysical Properties of a Series of Isostructural d6 Complexes Based on Cr0, MnI, and FeII, Journal of the American Chemical Society. (2024) 146. 10.1021/jacs.3c11580 PMC10885143 38334415

[50] Zoppellaro G. , Ostruszka R. , and Siskova K. , Engineered Protein-Iron and/or Gold-Protein-Iron Nanocomposites in Aqueous Solutions Upon UVA Light: Photo-Induced Electron Transfer Possibilities and Limitations, Journal of Photochemistry and Photobiology A: Chemistry. (2024) 450, 10.1016/j.jphotochem.2023.115415.

[51] Watanabe K. , Terao N. , Kii I. , Nakagawa R. , Niwa T. , and Hosoya T. , Indolizines Enabling Rapid Uncaging of Alcohols and Carboxylic Acids by Red Light-Induced Photooxidation, Organic Letters. (2020) 22, no. 14, 5434–5438, 10.1021/acs.orglett.0c01799. 32615768

[52] Li A. , Turro C. , and Kodanko J. J. , Ru (II) Polypyridyl Complexes as Photocages for Bioactive Compounds Containing Nitriles and Aromatic Heterocycles, Chemical Communications. (2018) 54, no. 11, 1280–1290, 10.1039/c7cc09000e, 2-s2.0-85041420195. 29323683 PMC5904840

[53] Gütlich P. , Goodwin H. A. , Hendrickson D. N. , and Pierpont C. G. , Valence Tautomeric Transition Metal Complexes, Spin Crossover in Transition Metal Compounds II. (2004) Springer, Berlin, 63–95.

[54] Frenking G. and Fröhlich N. , The Nature of the Bonding in Transition-Metal Compounds, Chemical Reviews. (2000) 100, no. 2, 717–774, 10.1021/cr980401l, 2-s2.0-0041464227. 11749249

[55] Shi G. , Monro S. , Hennigar R. et al., Ru (II) Dyads Derived From α-Oligothiophenes: A New Class of Potent and Versatile Photosensitizers for PDT, Coordination Chemistry Reviews. (2015) 282, 127–138, 10.1016/j.ccr.2014.04.012, 2-s2.0-84911386578.

[56] Swarnalatha K. , Rajkumar E. , Rajagopal S. , Ramaraj R. , Banu I. S. , and Ramamurthy P. , Proton Coupled Electron Transfer Reaction of Phenols With Excited State Ruthenium (II)-Polypyridyl Complexes, Journal of Physical Organic Chemistry. (2011) 24, no. 1, 14–21, 10.1002/poc.1696, 2-s2.0-78650876754.

[57] Sayala J. , Srivastava E. , Kumar P. , Shukla N. , Kumar A. , and Patra A. K. , Photocytotoxic Kinetically Stable Ruthenium (II)-N,N-Donor Polypyridyl Complexes of Oxalate With Anticancer Activity Against HepG2 Liver Cancer Cells, Dalton Transactions. (2024) 53. 10.1039/d3dt04058e 38349214

[58] Morais T. S. , Valente A. , Tomaz A. I. , Marques F. , and Garcia M. H. , Tracking Antitumor Metallodrugs: Promising Agents With the Ru (II)-and Fe (II)-Cyclopentadienyl Scaffolds, Future Medicinal Chemistry. (2016) 8, no. 5, 527–544, 10.4155/fmc.16.7, 2-s2.0-84966471252. 27096164

[59] Skoczynska A. , Lewinski A. , Pokora M. , Paneth P. , and Budzisz E. , An Overview of the Potential Medicinal and Pharmaceutical Properties of Ru (II)/(III) Complexes, International Journal of Molecular Sciences. (2023) 24, no. 11, 10.3390/ijms24119512. PMC10253973 37298471

[60] Verrucchi M. , Giacomazzo G. E. , Sfragano P. S. et al., Characterization of a Ruthenium (II) Complex in Singlet Oxygen-Mediated Photoelectrochemical Sensing, Langmuir. (2022) 39, no. 1, 679–689, 10.1021/acs.langmuir.2c03042. 36574357 PMC9835978

[61] Hennessey S. , González-Gómez R. , McCarthy K. et al., Enhanced Photostability and Photoactivity of Ruthenium Polypyridyl-Based Photocatalysts by Covalently Anchoring Onto Reduced Graphene Oxide, ACS Omega. (2024) 9, no. 12, 13872–13882, 10.1021/acsomega.3c08800. 38559923 PMC10976380

[62] Méndez-Lucio O. , Romo-Mancillas A. , Medina-Franco J. L. , and Castillo R. , Computational Study on the Inhibition Mechanism of Cruzain by Nitrile-Containing Molecules, Journal of Molecular Graphics and Modelling. (2012) 35, 28–35, 10.1016/j.jmgm.2012.01.003, 2-s2.0-84859793022. 22481076

[63] Müller P. , Meta M. , Meidner J. L. et al., Investigation of the Compatibility Between Warheads and Peptidomimetic Sequences of Protease Inhibitors—A Comprehensive Reactivity and Selectivity Study, International Journal of Molecular Sciences. (2023) 24, no. 8, 10.3390/ijms24087226. PMC10138721 37108388

[64] Prates J. L. B. , Lopes J. R. , Chin C. M. , Ferreira E. I. , Dos Santos J. L. , and Scarim C. B. , Discovery of Novel Inhibitors of Cruzain Cysteine Protease of *Trypanosoma cruzi* , Current Medicinal Chemistry. (2024) 31, no. 16, 2285–2308, 10.2174/0109298673254864230921090519. 37888814

[65] Brogi S. , Ibba R. , Rossi S. et al., Covalent Reversible Inhibitors of Cysteine Proteases Containing the Nitrile Warhead: Recent Advancement in the Field of Viral and Parasitic Diseases, Molecules. (2022) 27, no. 8, 10.3390/molecules27082561. PMC9029279 35458759

[66] Huang F. , Han X. , Xiao X. , and Zhou J. , Covalent Warheads Targeting Cysteine Residue: the Promising Approach in Drug Development, Molecules. (2022) 27, no. 22, 10.3390/molecules27227728. PMC9694382 36431829

[67] Lewiecki E. M. , Odanacatib, a Cathepsin K Inhibitor for the Treatment of Osteoporosis and Other Skeletal Disorders Associated With Excessive Bone Remodeling, Drugs. (2009) 12, no. 12, 799–809. 19943223

[68] Rocho F. R. , Bonatto V. , Lameiro R. F. , Lameira J. , Leitão A. , and Montanari C. A. , A Patent Review on Cathepsin K Inhibitors to Treat Osteoporosis (2011–2021), Expert Opinion on Therapeutic Patents. (2022) 32, no. 5, 561–573, 10.1080/13543776.2022.2040480. 35137661

[69] Gora J. and Latajka R. , Involvement of Cysteine Proteases in Cancer, Current Medicinal Chemistry. (2015) 22, no. 8, 944–957, 10.2174/0929867321666141106115624, 2-s2.0-84931263732. 25386822

[70] Jakoš T. , Pišlar A. , Jewett A. , and Kos J. , Cysteine Cathepsins in Tumor-Associated Immune Cells, Frontiers in Immunology. (2019) 10, 10.3389/fimmu.2019.02037, 2-s2.0-85072050781. PMC6724555 31555270

[71] Lalmanach G. , Kasabova-Arjomand M. , Lecaille F. , and Saidi A. , Cystatin M/E (Cystatin 6): A Janus-Faced Cysteine Protease Inhibitor With Both Tumor-Suppressing and Tumor-Promoting Functions, Cancers (Basel). (2021) 13, no. 8, 10.3390/cancers13081877. PMC8070812 33919854

[72] Kulkarni G. S. , Lilge L. , Nesbitt M. , Dumoulin-White R. J. , Mandel A. , and Jewett M. A. S. , A Phase 1b Clinical Study of Intravesical Photodynamic Therapy in Patients With *Bacillus calmette*-Guérin-Unresponsive Non-Muscle-Invasive Bladder Cancer, European Urology Open Science. (2022) 41, 105–111, 10.1016/j.euros.2022.04.015. 35813250 PMC9257636

[73] Bednarski P. J. , Korpis K. , Westendorf A. F. , Perfahl S. , and Grünert R. , Effects of Light-Activated Diazido-PtIV Complexes on Cancer Cells In Vitro, Philosophical Transactions of the Royal Society A: Mathematical, Physical and Engineering Sciences. (2013) 371, no. 1995, 10.1098/rsta.2012.0118, 2-s2.0-84887400140. 23776289

[74] Hess J. , Huang H. , Kaiser A. et al., Evaluation of the Medicinal Potential of Two Ruthenium (II) Polypyridine Complexes as One‐and Two‐Photon Photodynamic Therapy Photosensitizers, Chemistry-A European Journal. (2017) 23, no. 41, 9888–9896, 10.1002/chem.201701392, 2-s2.0-85025113722. 28509422

[75] Monro S. , Colon K. L. , Yin H. et al., Transition Metal Complexes and Photodynamic Therapy From a Tumor-Centered Approach: Challenges, Opportunities, and Highlights From the Development of TLD1433, Chemical Reviews. (2018) 119, no. 2, 797–828, 10.1021/acs.chemrev.8b00211, 2-s2.0-85054653173. 30295467 PMC6453754

[76] Qu F. , Lamb R. W. , Cameron C. G. et al., Singlet Oxygen Formation vs Photodissociation for Light-Responsive Protic Ruthenium Anticancer Compounds: The Oxygenated Substituent Determines Which Pathway Dominates, Inorganic Chemistry. (2021) 60, no. 4, 2138–2148, 10.1021/acs.inorgchem.0c02027. 33534562 PMC8006600

[77] Chen Y. , Bai L. , Zhang P. , Zhao H. , and Zhou Q. , The Development of Ru (II)-Based Photoactivated Chemotherapy Agents, Molecules. (2021) 26, no. 18, 10.3390/molecules26185679. PMC8465985 34577150

[78] He G. , He M. , Wang R. et al., A Near‐Infrared Light‐Activated Photocage Based on a Ruthenium Complex for Cancer Phototherapy, Angewandte Chemie International Edition. (2023) 62, no. 24, 10.1002/ange.202218768. 36890113

[79] Zhang Z. , He M. , Wang R. , Fan J. , Peng X. , and Sun W. , Development of Ruthenium Nanophotocages With Red or Near‐Infrared Light‐Responsiveness, ChemBioChem. (2023) 24, no. 24, 10.1002/cbic.202300606. 37837285

[80] Mishra R. , Saha A. , Chatterjee P. , Bhattacharyya A. , and Patra A. K. , Ruthenium (II) Polypyridyl-Based Photocages for an Anticancer Phytochemical Diallyl Sulfide: Comparative Dark and Photoreactivity Studies of Caged and Precursor Uncaged Complexes, Inorganic Chemistry. (2023) 62, no. 46, 18839–18855, 10.1021/acs.inorgchem.3c02038. 37930798

[81] Toupin N. , Development of New Photochemical Tools for Applications in Cancer Research and Enzymatic Signaling, 2022, Wayne State University.

[82] Filevich O. and Etchenique R. , RuBiGABA-2: A Hydrophilic Caged GABA With Long Wavelength Sensitivity, Photochemical & Photobiological Sciences. (2013) 12, no. 9, 1565–1570, 10.1039/c3pp25248e, 2-s2.0-84882417776. 23674097

[83] Pickens R. N. , Neyhouse B. J. , Reed D. T. , Ashton S. T. , and White J. K. , Visible Light-Activated CO Release and 1O2 Photosensitizer Formation With Ru (II), Mn (I) Complexes, Inorganic Chemistry. (2018) 57, no. 18, 11616–11625, 10.1021/acs.inorgchem.8b01759, 2-s2.0-85052879458. 30160480

[84] Whittemore T. J. , White T. A. , and Turro C. , New Ligand Design Provides Delocalization and Promotes Strong Absorption Throughout the Visible Region in a Ru (II) Complex, Journal of the American Chemical Society. (2018) 140, no. 1, 229–234, 10.1021/jacs.7b09389, 2-s2.0-85040344226. 29260869

[85] Schindler J. , Zhang Y. , Traber P. et al., A ππ∗ State Enables Photoaccumulation of Charges on a π-Extended Dipyridophenazine Ligand in a Ru (II) Polypyridine Complex, Journal of Physical Chemistry C. (2018) 122, no. 1, 83–95, 10.1021/acs.jpcc.7b08989, 2-s2.0-85038573003.

[86] Ogawa T. , Sinha N. , Pfund B. , Prescimone A. , and Wenger O. S. , Molecular Design Principles to Elongate the Metal-to-Ligand Charge Transfer Excited-State Lifetimes of Square-Planar Nickel (II) Complexes, Journal of the American Chemical Society. (2022) 144, no. 48, 21948–21960, 10.1021/jacs.2c08838. 36417782 PMC9732883

[87] Martinez-Alonso M. and Gasser G. , Ruthenium Polypyridyl Complex-Containing Bioconjugates, Coordination Chemistry Reviews. (2021) 434, 10.1016/j.ccr.2020.213736.

[88] van Rixel V. H. S. , Ramu V. , Auyeung A. B. et al., Photo-Uncaging of a Microtubule-Targeted Rigidin Analogue in Hypoxic Cancer Cells and in a Xenograft Mouse Model, Journal of the American Chemical Society. (2019) 141, no. 46, 18444–18454, 10.1021/jacs.9b07225. 31625740 PMC11774275

[89] Puttock E. , Transition Metal Complexes and Their Applications in Energy Conversion, 2017, Durham University.

[90] Mapley J. I. , Spectroscopic and Computational Investigations of Charge Transfer Excited States, 2022, University of Otago.

[91] Meijer M. S. , Carlos R. M. , Baptista M. S. , and Bonnet S. , Photomedicine With Inorganic Complexes: A Bright Future, Springer Handbook of Inorganic Photochemistry, 2022, Springer, Berlin, 1015–1033.

[92] Zhang C. , Yuan Q. , Zhang Z. , and Tang Y. , A pH-Responsive Drug Delivery System Based on Conjugated Polymer for Effective Synergistic Chemo-/Photodynamic Therapy, Molecules. (2023) 28, no. 1, 10.3390/molecules28010399. PMC9823741 36615594

[93] Algorri J. F. , Ochoa M. , Roldan-Varona P. , Rodriguez-Cobo L. , and Lopez-Higuera J. M. , Photodynamic Therapy: A Compendium of Latest Reviews, Cancers (Basel). (2021) 13, no. 17, 10.3390/cancers13174447. PMC8430498 34503255

[94] Nkune N. W. and Abrahamse H. , Anti-Hypoxia Nanoplatforms for Enhanced Photosensitizer Uptake and Photodynamic Therapy Effects in Cancer Cells, International Journal of Molecular Sciences. (2023) 24, no. 3, 10.3390/ijms24032656. PMC9916860 36768975

[95] Alvarez N. and Sevilla A. , Current Advances in Photodynamic Therapy (PDT) and the Future Potential of PDT-Combinatorial Cancer Therapies, International Journal of Molecular Sciences. (2024) 25, no. 2, 10.3390/ijms25021023. PMC10815790 38256096

[96] Jiang W. , Liang M. , Lei Q. , Li G. , and Wu S. , The Current Status of Photodynamic Therapy in Cancer Treatment, Cancers (Basel). (2023) 15, no. 3, 10.3390/cancers15030585. PMC9913255 36765543

[97] Clement S. , Guller A. , Mahbub S. B. , and Goldys E. M. , Oxygen-Carrying Polymer Nanoconstructs for Radiodynamic Therapy of Deep Hypoxic Malignant Tumors, Biomedicines. (2021) 9, no. 3, 10.3390/biomedicines9030322. PMC8005177 33810115

[98] Yang J.-K. , Kwon H. , and Kim S. , Recent Advances in Light-Triggered Cancer Immunotherapy, Journal of Materials Chemistry B. (2024) 12. 10.1039/d3tb02842a 38353138

[99] Medellin D. C. , Zhou Q. , Scott R. et al., Novel Microtubule-Targeting 7-Deazahypoxanthines Derived From Marine Alkaloid Rigidins With Potent In Vitro and In Vivo Anticancer Activities, Journal of Medicinal Chemistry. (2016) 59, no. 1, 480–485, 10.1021/acs.jmedchem.5b01426, 2-s2.0-84955250682. 26641132 PMC4950951

[100] Tsai C. N. , Tian Y.-H. , Shi X. et al., Experimental and DFT Characterization of Metal-to-Ligand Charge-Transfer Excited States of (Rutheniumammine)(Monodentate Aromatic Ligand) Chromophores, Inorganic Chemistry. (2013) 52, no. 17, 9774–9790, 10.1021/ic4016614, 2-s2.0-84961975545. 23952527

[101] Guo D. , Xu S. , Huang Y. et al., Platinum (IV) Complex-Based Two-in-One Polyprodrug for a Combinatorial Chemo-Photodynamic Therapy, Biomaterials. (2018) 177, 67–77, 10.1016/j.biomaterials.2018.05.052, 2-s2.0-85049349139. 29885587

[102] Karati D. , Meur S. , Mukherjee S. , and Roy S. , Revolutionizing Anticancer Treatment: Ruthenium-Based Nanoplatforms Pave New Paths, Coordination Chemistry Reviews. (2024) 519, 10.1016/j.ccr.2024.216118.

[103] Shi H. , Ward-Deitrich C. , Ponte F. , Sicilia E. , Goenaga-Infante H. , and Sadler P. J. , Photosubstitution and Photoreduction of a Diazido Platinum (IV) Anticancer Complex, Dalton Transactions. (2024) 53, no. 31, 13044–13054, 10.1039/d4dt01587h. 39028324

[104] Wei L. , Kushwaha R. , Sadhukhan T. et al., Dinuclear Tridentate Ru (II) Complex With Strong Near-Infrared Light-Triggered Anticancer Activity, Journal of Medicinal Chemistry. (2024) 67, no. 13, 11125–11137, 10.1021/acs.jmedchem.4c00624. 38905437

[105] Yang X. , Zhang Y. , Cao L. et al., Achieving Near-Infrared Emission Platinum (II) Complex by Introducing Dimerized Benzothiadiazole Unit, Optical Materials. (2022) 123, 10.1016/j.optmat.2021.111896.

[106] Gul A. , Ahmad M. , Ullah R. , Ullah R. , Kang Y. , and Liao W. , Systematic Review on Antibacterial Photodynamic Therapeutic Effects of Transition Metals Ruthenium and Iridium Complexes, Journal of Inorganic Biochemistry. (2024) 255, 10.1016/j.jinorgbio.2024.112523. 38489864

[107] Ballester F. J. , Hernández-García A. , Santana M. D. et al., Photoactivatable Ruthenium Complexes Containing Minimal Straining Benzothiazolyl-1,2,3-Triazole Chelators for Cancer Treatment, Inorganic Chemistry. (2024) 63. 10.1021/acs.inorgchem.3c04432 PMC11005040 38385171

[108] Spector D. , Bubley A. , Zharova A. et al., Light-Responsive Pt (IV) Prodrugs With Controlled Photoactivation and Low Dark Toxicity, ACS Applied Bio Materials. (2024) 7, no. 5, 3431–3440, 10.1021/acsabm.4c00345. 38697834

[109] D’Amato A. , Mariconda A. , Iacopetta D. et al., Complexes of Ruthenium (II) as Promising Dual-Active Agents Against Cancer and Viral Infections, Pharmaceuticals. (2023) 16, no. 12, 10.3390/ph16121729. PMC10747139 38139855

[110] Sheikh H. K. , Ortiz C. J. C. , Arshad T. , Padrón J. M. , and Khan H. , Advancements in Steroidal Pt (II) & Pt (IV) Derivatives for Targeted Chemotherapy (2000–2023), European Journal of Medicinal Chemistry. (2024) 271, 10.1016/j.ejmech.2024.116438. 38685141

[111] Zhong X. , Zhang Y. , and Wei J. , Recent Advances in Ruthenium(III) Complex-Loaded Nanomaterial for Enhanced Cancer Therapy Efficacy, Drug Development and Industrial Pharmacy. (2025) 51, 1–18, 10.1080/03639045.2025.2455428. 39836522

[112] Zhou Z. , Shi P. , Wang C. , Sun Y. , and Gao C. , Recent Updates in Nanoscale Delivery Systems of Platinum (IV) Antitumor Prodrugs, Coordination Chemistry Reviews. (2024) 508, 10.1016/j.ccr.2024.215774.

[113] Patra S. A. , Sahu G. , Das S. , and Dinda R. , Recent Advances in Mitochondria‐Localized Luminescent Ruthenium (II) Metallodrugs as Anticancer Agents, ChemMedChem. (2023) 18, no. 22, 10.1002/cmdc.202300397. 37772783

[114] Yang S.-Y. , Chen Y. , Kwok R. T. K. , Lam J. W. Y. , and Tang B. Z. , Platinum Complexes With Aggregation-Induced Emission, Chemical Society Reviews. (2024) 53. 10.1039/d4cs00218k 38712843

[115] Ang W. H. , Casini A. , Sava G. , and Dyson P. J. , Organometallic Ruthenium-Based Antitumor Compounds With Novel Modes of Action, Journal of Organometallic Chemistry. (2011) 696, no. 5, 989–998, 10.1016/j.jorganchem.2010.11.009, 2-s2.0-79952186235.

[116] Pervushin N. V. , Yapryntseva M. A. , Panteleev M. A. , Zhivotovsky B. , and Kopeina G. S. , Cisplatin Resistance and Metabolism: Simplification of Complexity, Cancers (Basel). (2024) 16, no. 17, 10.3390/cancers16173082. PMC11394643 39272940

[117] Xu L. , Kong X. , Li X. et al., Current Status of Novel Multifunctional Targeted Pt (IV) Compounds and Their Reductive Release Properties, Molecules. (2024) 29, no. 4, 10.3390/molecules29040746. PMC10892972 38398498

[118] Bonnet S. , Ruthenium-Based Photoactivated Chemotherapy, Journal of the American Chemical Society. (2023) 145, no. 43, 23397–23415, 10.1021/jacs.3c01135. 37846939 PMC10623564

[119] Li Y. , Liu X.-L. , Xu Q.-D. , Wei Z.-Q. , Wu X.-T. , and Sheng T.-L. , Influence of Electron-Donating Ability of Ligand and pH Value on MLCT Properties of Cyanido-Bridged Complexes, Inorganic Chemistry Communications. (2022) 140, 10.1016/j.inoche.2022.109446.

[120] Quintão S. V. M. , de Souza Bozzi A. , and Rocha W. R. , Exploring the Photophysics and Excited State Reactivity of [Ru (4, 4′-BTFMB) 2 (L)]^2+^ Complexes (L = Bpy, Phen, TAP) as Photodynamic Therapy Agents: A Theoretical Investigation, Inorganic Chemistry Frontiers. (2025) 12.

[121] Jang H. J. , Hopkins S. L. , Siegler M. A. , and Bonnet S. , Frontier Orbitals of Photosubstitutionally Active Ruthenium Complexes: An Experimental Study of the Spectator Ligands’ Electronic Properties Influence on Photoreactivity, Dalton Transactions. (2017) 46, no. 30, 9969–9980, 10.1039/c7dt01540b, 2-s2.0-85026832401. 28726891

[122] Morselli G. , Reber C. , and Wenger O. S. , Molecular Design Principles for Photoactive Transition Metal Complexes: A Guide for “Photo-Motivated” Chemists, Journal of the American Chemical Society. (2025) 147. 10.1021/jacs.5c02096 PMC11987026 40147007

[123] Kim D. , Dang V. Q. , and Teets T. S. , Improved Transition Metal Photosensitizers to Drive Advances in Photocatalysis, Chemical Science. (2024) 15, no. 1, 77–94, 10.1039/d3sc04580c. PMC10732135 38131090

[124] Murali R. , Biswas C. , Nayak S. K. et al., Influence of a Diketopyrrolopyrrole Spacer on the Ultrafast Nonlinear Optical Properties and Excited State Dynamics of Dimeric Zinc Porphyrin Molecules, Journal of Materials Chemistry C. (2025) 13, no. 2, 691–708, 10.1039/d4tc03281k.

[125] Maity S. , Kolay S. , Chakraborty S. , Devi A. , and Patra A. , A Comprehensive Review of Atomically Precise Metal Nanoclusters With Emergent Photophysical Properties Towards Diverse Applications, Chemical Society Reviews. (2025) 54. 10.1039/d4cs00962b 39670813

[126] Ciesienski K. L. , Hyman L. M. , Yang D. T. et al., A Photo‐Caged Platinum (II) Complex That Increases Cytotoxicity Upon Light Activation, 2010, Wiley Online Library.

[127] Chen Q.-B. , Zhou L.-Y. , Shi L.-X. et al., Platinum (IV) Complex-Loaded Nanoparticles With Photosensitive Activity for Cancer Therapy, Coordination Chemistry Reviews. (2022) 472, 10.1016/j.ccr.2022.214789.

[128] Zhao C.-L. , Qiao X. , Liu X.-M. et al., Rapid DNA Interstrand Cross-Linking of Pt (IV) Compound, European Journal of Pharmacology. (2022) 925, 10.1016/j.ejphar.2022.174985. 35489419

[129] Hall M. D. , Mellor H. R. , Callaghan R. , and Hambley T. W. , Basis for Design and Development of Platinum (IV) Anticancer Complexes, Journal of Medicinal Chemistry. (2007) 50, no. 15, 3403–3411, 10.1021/jm070280u, 2-s2.0-34547584200. 17602547

[130] Vo V. , Synthesis and Characterization of Novel Platinum (II) and Platinum (IV) Complexes Containing 4, 4′-Disubstituted--2, 2′-Bipyridine Ligands for the Treatment of Cancer, 2014.

[131] Ma J. , Wang Q. , Yang X. et al., Glycosylated Platinum (IV) Prodrugs Demonstrated Significant Therapeutic Efficacy in Cancer Cells and Minimized Side-Effects, Dalton Transactions. (2016) 45, no. 29, 11830–11838, 10.1039/c6dt02207c, 2-s2.0-84979231308. 27373800

[132] Phillips H. I. A. , Ronconi L. , and Sadler P. J. , Photoinduced Reactions of Cis, Trans, Cis‐[Ptiv (N3) 2 (OH) 2 (NH3) 2] With 1‐Methylimidazole, Chemistry-A European Journal. (2009) 15, no. 7, 1588–1596, 10.1002/chem.200802206, 2-s2.0-60749124450. 19140142 PMC2935676

[133] Mackay F. S. , Woods J. A. , Moseley H. et al., A Photoactivated Trans‐Diammine Platinum Complex as Cytotoxic as Cisplatin, Chemistry-A European Journal. (2006) 12, no. 11, 3155–3161, 10.1002/chem.200501601, 2-s2.0-33645698442. 16470886

[134] Shi H. , Imberti C. , and Sadler P. J. , Diazido Platinum (IV) Complexes for Photoactivated Anticancer Chemotherapy, Inorganic Chemistry Frontiers. (2019) 6, no. 7, 1623–1638, 10.1039/c9qi00288j, 2-s2.0-85068703879.

[135] Zhou D. , He S. , Cong Y. et al., A Polymer-(Multifunctional Single-Drug) Conjugate for Combination Therapy, Journal of Materials Chemistry B. (2015) 3, no. 24, 4913–4921, 10.1039/c5tb00576k, 2-s2.0-84953931659. 32262680

[136] Cong Y. , Wang Z. , He S. et al., Multifunctional Single-Drug Loaded Nanoparticles for Enhanced Cancer Treatment With Low Toxicity in Vivo, RSC Advances. (2016) 6, no. 24, 20366–20373, 10.1039/c5ra26372g, 2-s2.0-84958977018.

[137] He S. , Qi Y. , Kuang G. et al., Single-Stimulus Dual-Drug Sensitive Nanoplatform for Enhanced Photoactivated Therapy, Biomacromolecules. (2016) 17, no. 6, 2120–2127, 10.1021/acs.biomac.6b00353, 2-s2.0-84974803150. 27169722

[138] Song H. , Li W. , Qi R. et al., Delivering a Photosensitive Transplatin Prodrug to Overcome Cisplatin Drug Resistance, Chemical Communications. (2015) 51, no. 57, 11493–11495, 10.1039/c5cc03692e, 2-s2.0-84936966511. 26094840

[139] Xu S. , Zhu X. , Zhang C. , Huang W. , Zhou Y. , and Yan D. , Oxygen and Pt (II) Self-Generating Conjugate for Synergistic Photo-Chemo Therapy of Hypoxic Tumor, Nature Communications. (2018) 9, no. 1, 10.1038/s41467-018-04318-1, 2-s2.0-85047651889. PMC5967320 29795534

[140] Zhou X. , Liang H. , Jiang P. et al., Multifunctional Phosphorescent Conjugated Polymer Dots for Hypoxia Imaging and Photodynamic Therapy of Cancer Cells, Advanced Science. (2016) 3, no. 2, 10.1002/advs.201500155, 2-s2.0-85003451753. PMC5049659 27722081

[141] Huang Y.-Y. , Tian Y. , Liu X.-Q. et al., Luminescent Supramolecular Polymer Nanoparticles for Ratiometric Hypoxia Sensing, Imaging and Therapy, Materials Chemistry Frontiers. (2018) 2, no. 10, 1893–1899, 10.1039/c8qm00309b, 2-s2.0-85054213887.

[142] Cai H. , Wu X. , Jiang L. et al., Lysosome-Targeted Carbon Dots With a Light-Controlled Nitric Oxide Releasing Property for Enhanced Photodynamic Therapy, Chinese Chemical Letters. (2024) 35, no. 4, 10.1016/j.cclet.2023.108946.

[143] Juvekar V. , Lee D. J. , Park T. G. et al., Two-Photon Excitation Photosensitizers for Photodynamic Therapy: From Small-Molecules to Nano-Complex Systems, Coordination Chemistry Reviews. (2024) 506.

[144] Swaminathan S. , Haribabu J. , and Karvembu R. , From Concept to Cure: The Road Ahead for Ruthenium‐Based Anticancer Drugs, ChemMedChem. (2024) 19, no. 23, 10.1002/cmdc.202400435. 39374112

[145] Lilge L. , Roufaiel M. , Lazic S. et al., Evaluation of a Ruthenium Coordination Complex as Photosensitizer for PDT of Bladder Cancer: Cellular Response, Tissue Selectivity and In Vivo Response, Translational Biophotonics. (2020) 2, no. 1-2, 10.1002/tbio.201900032.

[146] Ravera M. , Gabano E. , McGlinchey M. J. , and Osella D. , Pt (IV) Antitumor Prodrugs: Dogmas, Paradigms, and Realities, Dalton Transactions. (2022) 51, no. 6, 2121–2134, 10.1039/d1dt03886a. 35015025

[147] Ye R. , Ke Z. , Tan C. , He L. , Ji L. , and Mao Z. , Histone‐Deacetylase‐Targeted Fluorescent Ruthenium (II) Polypyridyl Complexes as Potent Anticancer Agents, Chemistry-A European Journal. (2013) 19, no. 31, 10160–10169, 10.1002/chem.201300814, 2-s2.0-84881385613. 23828334

[148] Venkatesh V. and Sadler P. J. , Platinum (IV) Prodrugs, Metal Ions in Life Sciences. (2018) 18, no. 69. 10.1515/9783110470734-009 29394022

[149] Pickens R. N. , Synthesis and Characterization of Ruthenium and Manganese Mono-and BimetallicComplexes Towards the Photoactivated Release of Therapeutic Molecules, 2022, Ohio University.

[150] Abyar S. , Huang L. , Husiev Y. et al., Oxygen-Dependent Interactions Between the Ruthenium Cage and the Photoreleased Inhibitor in NAMPT-Targeted Photoactivated Chemotherapy, Journal of Medicinal Chemistry. (2024) 67, no. 13, 11086–11102, 10.1021/acs.jmedchem.4c00589. 38924492 PMC11247496

[151] Havrylyuk D. , Hachey A. C. , Fenton A. , Heidary D. K. , and Glazer E. C. , Ru (II) Photocages Enable Precise Control Over Enzyme Activity With Red Light, Nature Communications. (2022) 13, no. 1, 10.1038/s41467-022-31269-5. PMC9233675 35752630

[152] Kuznetsov K. M. , Cariou K. , and Gasser G. , Two in One: Merging Photoactivated Chemotherapy and Photodynamic Therapy to Fight Cancer, Chemical Science. (2024) 15, no. 43, 17760–17780, 10.1039/d4sc04608k. 39464604 PMC11499979

[153] Shah N. , Karnik R. , Darji S. , Devkar R. V. , Banerjee D. , and Chakraborty D. , Photoactive Ruthenium Polypyridine Cage Incorporating Doxorubicin: Synthesis, Photorelease, and Photocytotoxicity of Doxorubicin With Blue Light, Inorganic Chemistry Communications. (2024) 167, 10.1016/j.inoche.2024.112573.

[154] Dunkel P. , Photoremovable Protecting Groups, Encyclopedia. (2022) 2, no. 3, 1225–1236, 10.3390/encyclopedia2030082.

[155] Aranda E. S. , Molecular Photoswitches: Towards the Rational Design of Donor-Acceptor Stenhouse Adducts and Photoswitchable Transmembrane Peptides, 2021, Universidad de la Rioja.

[156] De Vos D. , Gadde K. , and Maes B. U. W. , Emerging Activation Modes and Techniques in Visible-Light-Photocatalyzed Organic Synthesis, Synthesis. (2023) 55, no. 02, 193–231, 10.1055/a-1946-0512.

[157] Liu J. , Kang W. , and Wang W. , Photocleavage‐Based Photoresponsive Drug Delivery, Photochemistry and Photobiology. (2022) 98, no. 2, 288–302, 10.1111/php.13570. 34861053

[158] Panda B. , The Recent Developments and Applications of Photoremovable Protecting Groups in Organic Chemistry, Current Chinese Chemistry. (2022) 2, 10.2174/2666001602666220202142858.

[159] Dong M. , Babalhavaeji A. , Samanta S. , Beharry A. A. , and Woolley G. A. , Red-Shifting Azobenzene Photoswitches for In Vivo Use, Accounts of Chemical Research. (2015) 48, no. 10, 2662–2670, 10.1021/acs.accounts.5b00270, 2-s2.0-84945901276. 26415024

[160] Jerca F. A. , Jerca V. V. , and Hoogenboom R. , Advances and Opportunities in the Exciting World of Azobenzenes, Nature Reviews Chemistry. (2022) 6, no. 1, 51–69, 10.1038/s41570-021-00334-w. 37117615

[161] Beharry A. A. and Woolley G. A. , Azobenzene Photoswitches for Biomolecules, Chemical Society Reviews. (2011) 40, no. 8, 4422–4437, 10.1039/c1cs15023e, 2-s2.0-79960476905. 21483974

[162] Zhu M. and Zhou H. , Azobenzene-Based Small Molecular Photoswitches for Protein Modulation, Organic & Biomolecular Chemistry. (2018) 16, no. 44, 8434–8445, 10.1039/c8ob02157k, 2-s2.0-85056473660. 30375620

[163] Chen Y.-C. , Huang X.-C. , Luo Y.-L. , Chang Y.-C. , Hsieh Y.-Z. , and Hsu H.-Y. , Non-Metallic Nanomaterials in Cancer Theranostics: A Review of Silica-and Carbon-Based Drug Delivery Systems, Science and Technology of Advanced Materials. (2013) 14, no. 4, 10.1088/1468-6996/14/4/044407, 2-s2.0-84884892021. PMC5090318 27877592

[164] Chung K.-Y. , Uddin A. , and Page Z. A. , Record Release of Tetramethylguanidine Using a Green Light Activated Photocage for Rapid Synthesis of Soft Materials, Chemical Science. (2023) 14, no. 39, 10736–10743, 10.1039/d3sc04130a. 37829029 PMC10566505

[165] Dunbar M. N. , Steinke S. J. , Piechota E. J. , and Turro C. , Differences in Photophysical Properties and Photochemistry of Ru (II)-Terpyridine Complexes of CH3CN and Pyridine, Journal of Physical Chemistry A. (2024) 128, no. 3, 599–610, 10.1021/acs.jpca.3c07432. 38227956

[166] Sharma M. , Bakshi A. K. , Mittapelly N. et al., Recent Updates on Innovative Approaches to Overcome Drug Resistance for Better Outcomes in Cancer, Journal of Controlled Release. (2022) 346, 43–70, 10.1016/j.jconrel.2022.04.007. 35405165

[167] Hadi M. , Qutaiba B. , Allela O. et al., Recent Advances in Various Adeno-Associated Viruses (AAVs) as Gene Therapy Agents in Hepatocellular Carcinoma, Virology Journal. (2024) 21, no. 1, 10.1186/s12985-024-02286-1. PMC10785434 38216938

[168] Szymaszek P. , Tyszka-Czochara M. , and Ortyl J. , Application of Photoactive Compounds in Cancer Theranostics: Review on Recent Trends From Photoactive Chemistry to Artificial Intelligence, Molecules. (2024) 29, no. 13, 10.3390/molecules29133164. PMC11243723 38999115

[169] Li X. , Peng X. , Zoulikha M. et al., Multifunctional Nanoparticle-Mediated Combining Therapy for Human Diseases, Signal Transduction and Targeted Therapy. (2024) 9, no. 1, 10.1038/s41392-023-01668-1. PMC10758001 38161204

[170] Tran T. H. and Tran T. T. P. , Current Status of Nanoparticle-Mediated Immunogenic Cell Death in Cancer Immunotherapy, International Immunopharmacology. (2024) 142, no. Pt A, 10.1016/j.intimp.2024.113085. 39276455

[171] Tang F. , Ding A. , Xu Y. et al., Gene and Photothermal Combination Therapy: Principle, Materials, and Amplified Anticancer Intervention, Small. (2024) 20, no. 6, 10.1002/smll.202307078. 37775950

[172] Lin J. , Wang X. , Ni D. , Chen Y. , Chen C. , and Liu Y. , Combinational Gene Therapy Toward Cancer With Nanoplatform: Strategies and Principles, ACS Materials. (2023) 3, no. 6, 584–599, 10.1021/acsmaterialsau.3c00035. PMC10636764 38089659

[173] Yadav K. , Sahu K. K. , Gnanakani S. P. E. et al., Biomedical Applications of Nanomaterials in the Advancement of Nucleic Acid Therapy: Mechanistic Challenges, Delivery Strategies, and Therapeutic Applications, International Journal of Biological Macromolecules. (2023) 241, 10.1016/j.ijbiomac.2023.124582. 37116843

[174] Deng Z. , Wang N. , Liu Y. et al., A Photocaged, Water-Oxidizing, and Nucleolus-Targeted Pt (IV) Complex With a Distinct Anticancer Mechanism, Journal of the American Chemical Society. (2020) 142, no. 17, 7803–7812, 10.1021/jacs.0c00221. 32216337

[175] Bonsall S. , Hubbard S. , Jithin U. et al., Water-Soluble Truncated Fatty Acid-Porphyrin Conjugates Provide Photo-Sensitizer Activity for Photodynamic Therapy in Malignant Mesothelioma, Cancers (Basel). (2022) 14, no. 21, 10.3390/cancers14215446. PMC9654571 36358864

[176] Karges J. , Clinical Development of Metal Complexes as Photosensitizers for Photodynamic Therapy of Cancer, Angewandte Chemie International Edition. (2022) 61, no. 5, 10.1002/anie.202112236. 34748690

[177] Mallidi S. , Anbil S. , Bulin A.-L. , Obaid G. , Ichikawa M. , and Hasan T. , Beyond the Barriers of Light Penetration: Strategies, Perspectives and Possibilities for Photodynamic Therapy, Theranostics. (2016) 6, no. 13, 2458–2487, 10.7150/thno.16183, 2-s2.0-84999635721. 27877247 PMC5118607

[178] Gao J. , Chen Z. , Li X. et al., Chemiluminescence in Combination With Organic Photosensitizers: Beyond the Light Penetration Depth Limit of Photodynamic Therapy, International Journal of Molecular Sciences. (2022) 23, no. 20, 10.3390/ijms232012556. PMC9604449 36293406

[179] Garland M. J. , Cassidy C. M. , Woolfson D. , and Donnelly R. F. , Designing Photosensitizers for Photodynamic Therapy: Strategies, Challenges and Promising Developments, Future Medicinal Chemistry. (2009) 1, no. 4, 667–691, 10.4155/fmc.09.55, 2-s2.0-77953464531. 21426032

[180] Pervaiz S. and Olivo M. , Art and Science of Photodynamic Therapy, Clinical and Experimental Pharmacology and Physiology. (2006) 33, no. 5‐6, 551–556, 10.1111/j.1440-1681.2006.04406.x, 2-s2.0-33745194617. 16700893

[181] Sinha L. , Elliott J. T. , Hasan T. , Pogue B. W. , Samkoe K. S. , and Tichauer K. M. , Early Photosensitizer Uptake Kinetics Predict Optimum Drug-Light Interval for Photodynamic Therapy, Optical Methods for Tumor Treatment and Detection: Mechanisms and Techniques in Photodynamic Therapy XXIV, 2015, SPIE, Bellingham, WA, 104–111.

[182] Veld R. V. , Heuts J. , Ma S. , Cruz L. J. , Ossendorp F. A. , and Jager M. J. , Current Challenges and Opportunities of Photodynamic Therapy Against Cancer, Pharmaceutics. (2023) 15, no. 2, 10.3390/pharmaceutics15020330. PMC9965442 36839652

[183] Rodrigues J. A. and Correia J. H. , Enhanced Photodynamic Therapy: A Review of Combined Energy Sources, Cells. (2022) 11, no. 24, 10.3390/cells11243995. PMC9776440 36552759

[184] Hong L. , Li J. , Luo Y. et al., Recent Advances in Strategies for Addressing Hypoxia in Tumor Photodynamic Therapy, Biomolecules. (2022) 12, no. 1, 10.3390/biom12010081. PMC8774200 35053229

[185] Szymaszek P. , Tyszka-Czochara M. , and Ortyl J. , Iridium (III) Complexes as Novel Theranostic Small Molecules for Medical Diagnostics, Precise Imaging at a Single Cell Level and Targeted Anticancer Therapy, European Journal of Medicinal Chemistry. (2024) 276, 10.1016/j.ejmech.2024.116648. 38968786

[186] An Y. , Xu D. , Wen X. , Chen C. , Liu G. , and Lu Z. , Internal Light Sources‐Mediated Photodynamic Therapy Nanoplatforms: Hope for the Resolution of the Traditional Penetration Problem, Advanced Healthcare Materials. (2024) 13, no. 1, 10.1002/adhm.202301326. 37413664

[187] Zhao Y. , Liu X. , Liu X. et al., Combination of Phototherapy With Immune Checkpoint Blockade: Theory and Practice in Cancer, Frontiers in Immunology. (2022) 13, 10.3389/fimmu.2022.955920. PMC9478587 36119019

